# Specially Designed Polyaniline/Polypyrrole Ink for a Fully Printed Highly Sensitive pH Microsensor

**DOI:** 10.1021/acsami.1c08043

**Published:** 2021-07-06

**Authors:** Miguel Zea, Robert Texidó, Rosa Villa, Salvador Borrós, Gemma Gabriel

**Affiliations:** † Instituto de Microelectrónica de Barcelona IMB-CNM (CSIC), Campus Universitat Autònoma de Barcelona, 08193 Cerdanyola del Vallès, Barcelona, Spain; ‡ PhD in Electrical and Telecommunication Engineering, Universitat Autonoma de Barcelona (UAB), Barcelona, Spain; § Grup d’Enginyeria de Materials, Institut Químic de Sarrià-Universitat Ramon Llull, vía Augusta 390, 08017 Barcelona, Spain; ∥ CIBER de Bioingeniería, Biomateriales y Nanomedicina (CIBER-BBN), Zaragoza, Spain

**Keywords:** pH sensor, polymeric ink, polyaniline, polypyrrole, inkjet printing

## Abstract

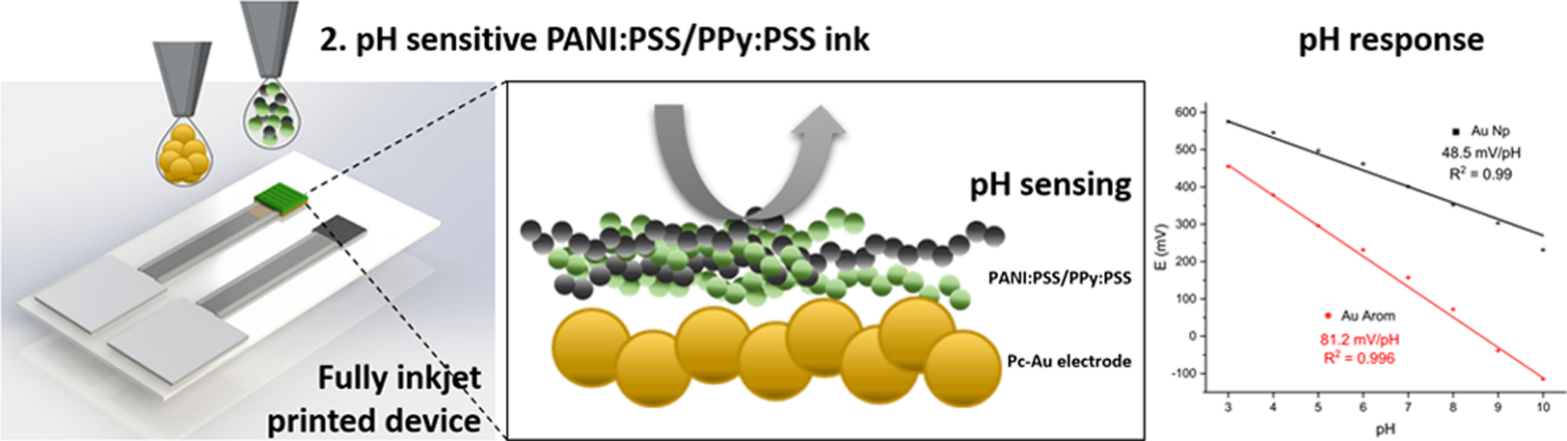

pH sensing for healthcare applications requires sensors with mechanically stable materials of high sensitivity and high reproducibility combined with low-cost fabrication technologies. This work proposes a fully printed pH sensor based on a specially formulated conducting polymer deposited on a microelectrode in a flexible substrate. A formulation, which combined polyaniline (PANI) and polypyrrole (PPy) with integrated polyelectrolyte poly(sodium 4-styrenesulfonate) (PSS), was specially prepared to be printed by inkjet printing (IJP). The sensor has good sensitivity in the physiological region (pH 7–7.5) key for the healthcare biosensor. This mixture printed over a commercial gold ink, which has a singular chemical functionalization with phthalocyanine (Pc), increased the sensor sensitivity, showing an excellent reproducibility with a linear super-Nernstian response (81.2 ± 0.5 mV/pH unit) in a wide pH range (pH 3–10). This new ink together with the IJP low-cost technique opens new opportunities for pH sensing in the healthcare field with a single device, which is disposable, highly sensitive, and stable in the whole pH range.

## Introduction

pH is a key indicator for many biochemical processes and for this reason, pH sensors have received considerable attention for monitoring human healthcare due to their versatility, possibility of real-time measurements, and quantitative results.^1^ Possible areas of interest can be found in the continuous monitoring of blood^2,3^ and sweat^4,5^ and the determination of the pH of tumors^6^ as the chemodynamic therapy heavily depends on acidic chemical environment pH measurements that can determine the effectiveness of this treatment. Even though the pH sensor is a broadly studied one, for all of the abovementioned applications, integrated pH sensors must fulfill strict requirements that are technologically unresolved to date. The most relevant points where the technology still faces important challenges to be accomplished are^7^ (1) flexibility, to be adapted to body tissues; (2) the need for miniaturized pH sensors that are minimally invasive; (3) good performance such as good stability, sensitivity, and response time, to allow monitoring for definite time; and (4) accuracy at neutral pH, especially interesting for physiological measurements.

Most of these pH sensors are designed for single use and for this reason, their fabrication technology must be easy to allow scaling up and production of a wide range of devices and at a reasonable cost. In particular, the healthcare field is growing toward a promising platform for personal wearable electronic and flexible biochemical sensors, which are envisaged to replace bulky and costly medical instruments for healthcare monitoring or at least, to complement the laboratory-based devices, expanding the areas of application. One of the most important digital fabrication techniques in flexible electronics for the fabrication of sensors is inkjet printing (IJP). In the last few years, flexible electronics has grown exponentially in application fields such as healthcare and industry, and the market is expected to reach USD 87.21 billion by 2024.^8^ It is an additive manufacturing and noncontact approach that allows the maskless deposition of functional materials in small drop volumes on a wide range of substrates.^9^ These singular features are of significant interest for the fabrication of biochemical sensors due to their simple implementation and scalability to high-volume manufacturing. Although IJP cannot be considered as a replacement for conventional silicon fabrication techniques, it is a reliable technique for the development of sensors with the abovementioned requirements, namely, low cost, disposable, robust, and miniaturized^10^ in novel low-cost flexible substrates such as a broad range of plastics and even paper with real applicability in the future.^11^

As for pH sensors fabricated by IJP reported to date, due to simplicity and the possibility of miniaturization, all of those reported have been electrochemical sensors based on potentiometric techniques.^12^ The printed metallic electrodes have been functionalized by depositing or growing some of the most representative pH-sensitive materials, usually oxides, polymers, and biomolecules. The sensing materials that have a pH-dependent potential over printed metallic inks proved until now are electrochemically grown iridium oxide,^13,14^ palladium oxide grown over a printed palladium ink (Pd/PdO),^14,15^ and electropolymerized polyaniline (PANI) polymers.^16^

Conducting polymers (CPs) are ideally suited for pH-sensing applications^17,18^ because they not only exhibit high conductivity and electroactivity but can also be used as a general matrix and can be further modified with other compounds to increase the pH range.^19^ Although many polymers have been studied,^20^ the most popular polymer-film-coated electrodes reported are the ones using electropolymerized pyrrole and aniline.^21,22^ For this work, CPs have been considered as the optimal candidate among all of the range of materials used for pH sensors because they allow the formulation of a suitable ink with adequate rheological properties to be printed by the IJP technique.

In this work, we propose a printable pH-sensitive CP that allows the first fully printed polymer-based pH sensor reported until now. We develop a new water-based ink formulation based on a mixture of PANI:PSS/PPy:PSS that gives a high stability and super-Nernstian sensitivity (81 mV/pH) to the final performance of the pH sensor. This mixture of polymers pays special attention to neutral pH (pH values 7–7.5), key for the healthcare biosensor and monitoring applications. To obtain physiological measurements in this pH interval is still a challenge due to the high precision and resolution required in this narrow range as well as the difficulty to obtain a stable measure without pronounced potential drifts. The proposed ink displayed a good linearity for the range of pH 3–10, solving the common CP drawback of a good performance at a physiological range of pH. Therefore, the inks and the fabrication approach herein described can open new ways for the mass production of miniaturized pH sensors for healthcare applications fully compatible with large-scale production methods.

## Experimental Section

### Materials and Chemicals

For the development of the microelectrodes, we used four commercially available inks. A low-curing gold colloidal ink (Au–Pc) (DryCure Au-J 1010B from Colloidal Ink Co., Ltd., Japan), low-curing gold nanoparticle ink (Au-Np) (Au-LT-20 from Fraunhofer IKTS, Germany), silver nanoparticle ink (Dupont-PE410), and SU-8 ink (2002 from MicroChem). All of the ink formulations were printed with a drop-on-demand Dimatix Materials Printer. Hydrochloric acid (HCl, 0.1 M) was used for the chlorination of the printed Ag electrode and potassium chloride (KCl, 0.1 M) for testing the pseudoreference electrode (pRE). Polyethylene naphthalate of 125 μm (PEN, TeonexQ65HA DuPont Teijin Films) was used as a substrate to print the microelectrodes. Commercial buffer solutions of pH 3–10 (Panreac), HCl and sodium hydroxide (NaOH) were used for the pH calibration (all reagents from Sigma-Aldrich).

### Polyaniline and Polypyrrole (PPy) Synthesis

The materials used in the synthesis of water-dispersible CP suspensions consisted of aniline (≥99,5%, Sigma-Aldrich, San Louis, MO), pyrrole (≥99%, Sigma-Aldrich, San Louis, MO), ammonium persulfate (≥98%, Fluka Bucharest, Romania), and poly(sodium 4-styrenesulfonate) (PSS) solution (*M*_w_ 70 000, 30 wt %; *M*_w_ 200 000, 30 wt %) purchased from Sigma-Aldrich (San Louis, MO).

### PANI and PPy Ink Formulation

PANI:PSS and PPy:PSS were formulated adding the proper amount of MilliQ water (MQ), Triton X100 as surfactant, and glycerol and dimethyl sulfoxide (DMSO) as the conductor promoter. Details of all of the prepared formulations are presented in Table S1.

### Ink Characterization: Dynamic Light Scattering (DLS)

The particle size distribution of the suspensions was determined by dynamic light scattering in diluted samples (1:10) using a Zetasizer ZEN3600 (Malvern Panalytical, Malvern, U.K.).

### Fourier Transform Infrared (FTIR) Spectra

PANI:PSS and PPy:PSS IR spectra were obtained using an IR spectrometer (Nicolet iS10, Thermo Fisher Scientific). For sample preparation, the suspension was lyophilized and then combined with potassium bromide (KBr) to be compressed between platens to form pellet for analysis.

### Microscopic Images

Field-emission scanning electron microscopy (FESEM) images were taken to observe the nanostructure with a field-emission Zeiss Merlin FESEM (Oberkochen, Germany). The film of the samples was prepared depositing a droplet of the suspension on a silicon substrate and dried on a vacuum drier at room temperature.

### Fabrication of Solid-State pH Sensor

**Figure 1:**
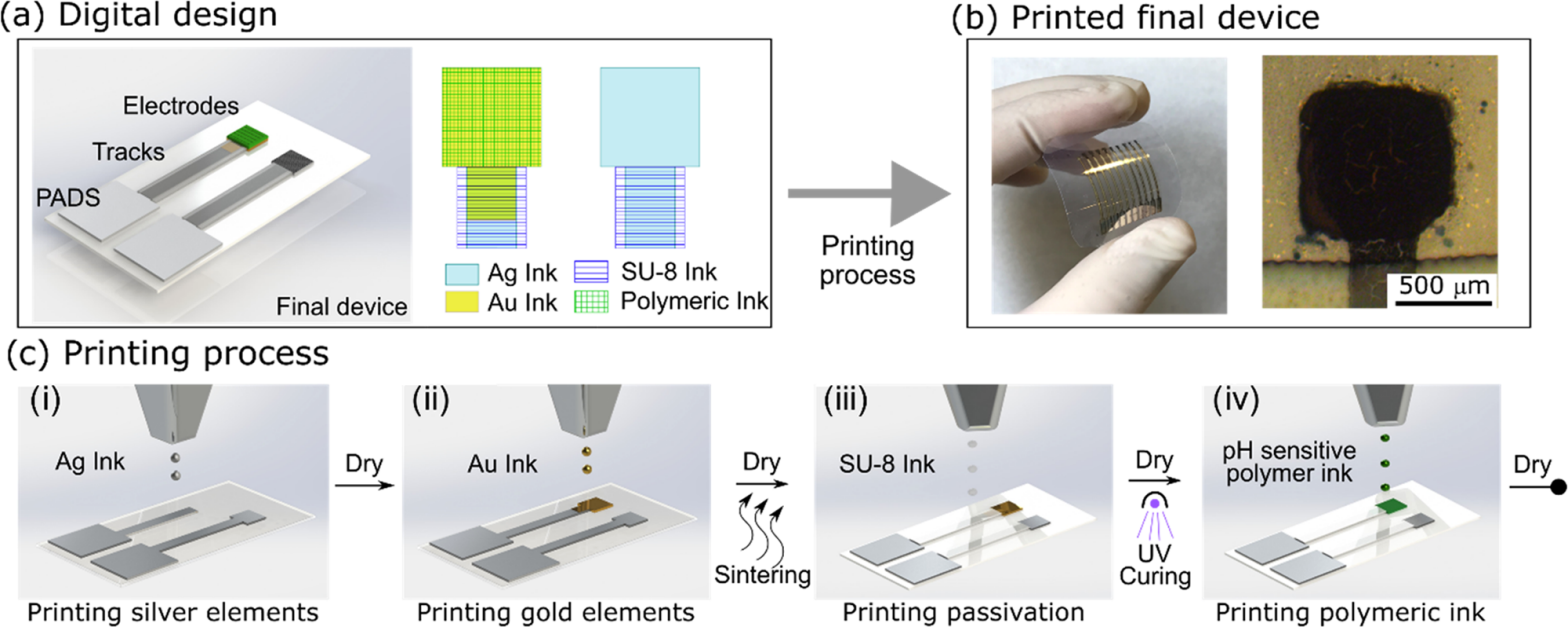
(a) Digital design of the pH sensor. (b) Final printed platform with 9 IE and 1 pRE and microscopic image of the printed polymeric electrodes. (c) Inkjet printing steps: (i) printing of Ag RE, tracks, and pads, followed by a dry step; (ii) printing of the Au IE, followed by a dry and thermal sintering of both metallic Au and Ag layers; (iii) printing of the dielectric SU-8, drying, and UV cross-linking; and (iv) printing of CP inks, followed by a drying step.

The pH sensor structure was fabricated with IJP technology, using the same approach that we reported in our previous work.^13,23^ Basically, a drop-on-demand Dimatix Material Printer (DMP 2831 for Fujifilm Dimatix, Inc.) was used with the 10 pL printhead. The sensor structure was digitally designed using CleWin 5 software (Figure 1a) and then exported to BMP to load in the Dimatix Bitmap editor. The printing process was carried out in standard laboratory conditions without particle filter, temperature, and humidity control. Microelectrodes and CP inks (PANI:PSS, PPy:PSS, and PANI:PSS/PPy:PSS) were printed on the PEN substrate, as detailed in Figure 1c. The complete fabrication of the pH sensor can be easily understood as two steps: The printing process of the metallic microelectrodes and the modification of electrodes. In the first step (c-i), the silver (Ag) elements were first printed: the reference electrode (RE) of 800 × 800 μm^2^, tracks, and pads with a drop spacing (DS) of 20 μm followed by a dry step at 80 °C for 15 min. Then, the Au-Np ink was printed (c-ii) to obtain a 1 mm^2^ indicator electrode (IE), with a DS of 15 μm, the platen temperature was set to 40 °C, and the printhead temperature was set to 30 °C. Au elements were dried as before, and both inks were finally sintered in the oven at 140 °C for 30 min. Then, the dielectric ink SU-8 was printed (c-iii) on Ag tracks to isolate and define the active electrode area and pads with a DS of 15 μm. First, a soft bake on a hot plate at 100 °C for 5 min, followed by a UV treatment for 30 s to polymerize this layer by polymer cross-linking. For the fabrication of microelectrodes with the other Au ink Au–Pc, the DS used was 25 μm and the platen and printhead temperature was set to 35 °C.

The second step consisted of the printing process of the pH-sensitive polymer ink over the Au microelectrode (c-iv) and the chlorination of the Ag RE. Initially, the CP inks were sonified for 5 min to uniformly disperse the solid content. Then, the inks were filtered through a polar ultrafilter of a 0.45 μm pore size and introduced into the ink cartridge. To achieve a good pattern definition substrate, the cartridge nozzles were heated at 40 and 35 °C. Three layers of CP inks were printed wet-on-wet on top of the Au IE with a DS of 10 μm and dried for 15 min at 120 °C. The Ag RE was chlorinated by cyclic voltammetry (detailed in Supporting Information (SI) Figure S1) in 0.1 M HCl, scanning the potential from 0 to 0.2 V versus Ag/AgCl commercial reference electrode at 20 mV/s to obtain a stable Ag/AgCl pseudoreference electrode (pRE). A final printed platform on a PEN substrate with nine IE and one pRE and a microscopic image of the printed CP electrodes can be observed in Figure 1b.

### Morphological Characterization of Printed CP

The printed polymeric films were analyzed using an optical microscope (DM4000M LEICA, Germany), a profilometer (KLA Tencor P-15), and a scanning electron microscope (SEM, Auriga40 from Carl Zeiss, Germany).

### pH Measurements

The open-circuit potential (OCP) of the inkjet-printed electrodes was measured by a PalmSens3 electrochemical station (De Indruk, Netherland). Measurements were carried out in various commercial pH buffer solutions ranging from pH 3 to 10. Sequential immersion in buffer solutions was followed by a cleaning step with deionized water to avoid cross-contamination. For each test, the electrodes were immersed in the buffer for 30 s and the potential between IE and pRE was recorded. The base–acid titration was done from basic (NaOH) to acidic (HCl) standardized solutions with concentrations of 0.01 mM, 0.1 mM, 0.001 M, 0.01 M, 0.1 M, and 1 M, which was magnetically stirred. Additionally, the pH value was recorded with a commercial pH-meter (GLP22, Crison) in both experiments.

## Results and Discussion

### Synthesis and Characterization of Printable Polymeric pH-Sensitive Inks

**Figure 2:**
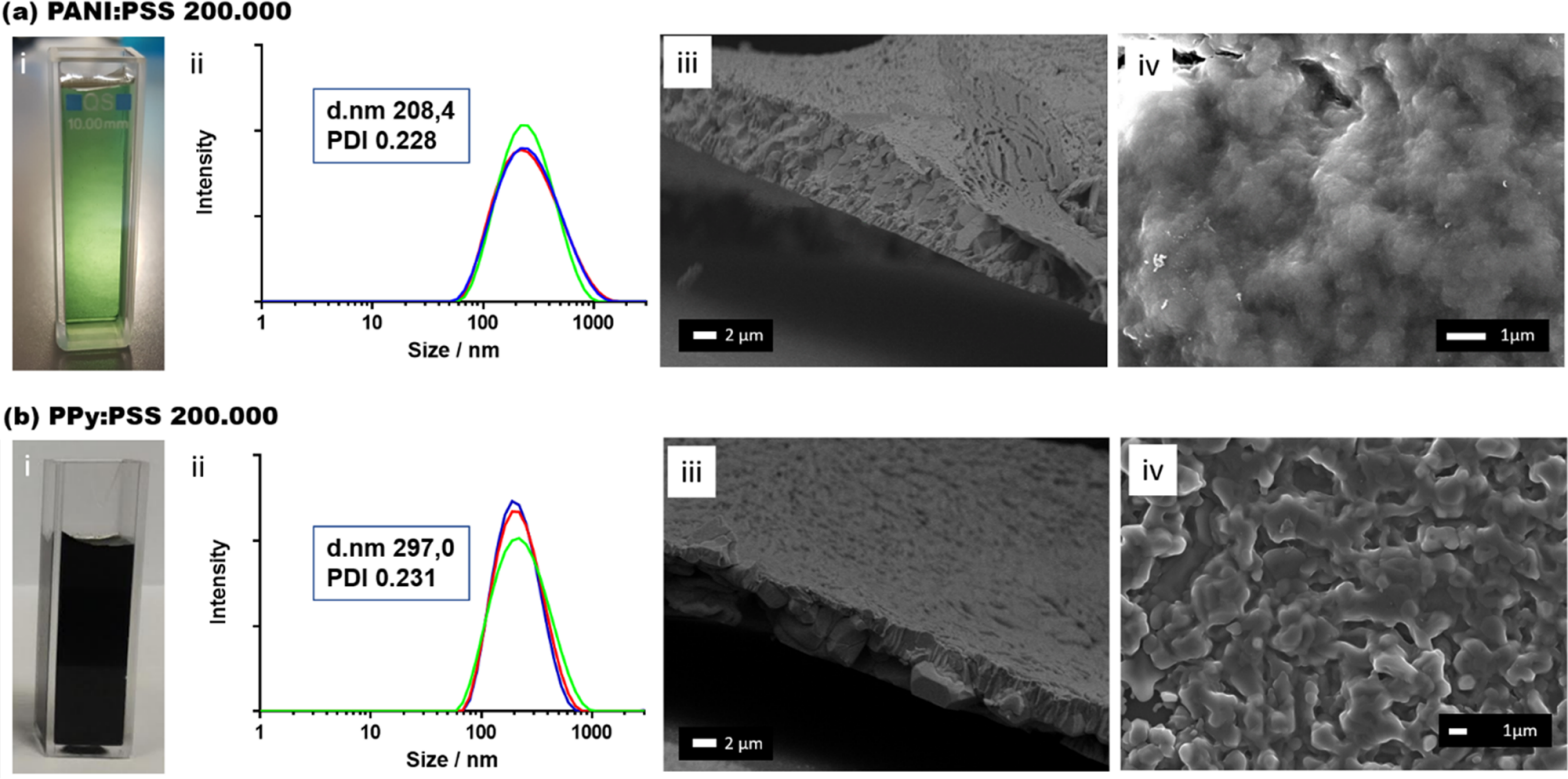
Characterization of CP suspensions. (a) PANI:PSS(II) suspension and (b) PPy:PSS(II) suspension: (i) solutions, (ii) particle size distribution, (iii) FESEM profile image, and (iv) FESEM film image.

To obtain the CP inks for IJP, water-dispersible particles of PANI and PPy were synthesized through the electrostatic interaction suspension method as described in the Experimental Section. This method is especially suitable to obtain CP suspensions with the ability to form a continuous conductive film as it has been previously reported for PANI^24,25^ and PPy.^26−28^ The distinctive feature of this procedure consists of involving electrostatic forces produced between monomers and polyelectrolytes to allow obtaining stable suspensions during the oxidative polymerization. This allows obtaining a controlled size distribution to fulfill one of the most restrictive IJP requirements. The supporting polyelectrolyte used was PSS solution as the charge-balancing dopant during the polymerization. Considering the application of the suspensions is an IJP ink, we studied the influence of the molecular weight of PSS which takes a critical role both in the properties of the ink (size distribution, stability) and in the properties of the printed film. In the current literature, it has been reported that high PSS molecular weight presents some advantages in terms of superior electrical and electrochemical performance.^29,30^ However, there are very few references about the effect of PSS molecular weight on the size distribution, suspension stability, and film formation properties of PANI:PSS and PPy:PSS suspensions. With this in mind, four different suspensions were prepared, (PANI:PSS(I), PANI:PSS(II), PPy:PPS(I), and PPy:PPS(II)), using PSS with two different molecular weights 70 000 Da for (I) suspensions and 200 000 Da for (II). After the synthesis, stable suspensions of both PANI (green color, Figure 2a-i) and PPy (black color, Figure 2b-i) were obtained for the two PSS used. Obtaining PANI:PSS and PPy:PSS suspensions through electrostatic interaction synthesis was confirmed through IR analysis as detailed in Supporting Information Figure S2a,b. None of the suspensions obtained presented signs of precipitation after 6 months at room temperature. Size distribution of the PANI:PSS and PPy:PSS formulations was studied through DLS. We observed that PANI:PSS and PPy:PSS suspensions presented a monodisperse size distribution in all cases, regardless of the monomer used or the PSS molecular weights. Considering the dependence of PANI polymer on the pH media, the synthesis of PANI:PSS suspensions was optimized at pH 2 to maximize the number of PANI:PSS particles (data shown in Figure S3). The particle size average values were around 200 nm, as shown in Figure 2a-ii for PANI:PSS(II) and in Figure 2b-ii for PPy:PSS(II) (values for PANI:PSS(I) and PPy:PSS(I) are shown in Supporting Information Figure S4). The ζ-potential of the suspensions was also measured, obtaining a value around −20 mV for PSS of *M*_w_ 70 000 and around −40 mV for the PSS of 200 000 (data shown in Figure S5). The mean value combined with particle size distribution (PDI) values around 0.2 obtained for each suspension fulfilled the requirements of IJP to avoid the clogging of the printer nozzle. All of the suspensions were correctly filtered using a 0.2 μm poly(tetrafluoroethylene) (PTFE) filter before each printing for the elimination of any possible higher particles to prevent the nozzle clogging.

After filtering, the suspensions were able to form a continuous film on a silicon substrate after air drying. FESEM images revealed a continuous structure with a well-defined layer thickness of approximately 5 μm for PANI:PSS (Figure 2a-iii) and 7 μm for PPy:PSS (Figure 2b-iii) for all of the film suspensions studied (Supporting Information Figure S6). No significant differences were observed in the morphology for PANI:PSS or PPy:PSS samples where a different molecular weight PSS was used during their synthesis (Figure 2a-iv,b-iv for *M*_w_ 200 000 PSS). As can be seen, both images present a globular morphology due to the interaction between the oxidant and the structuring agent during polymerization.^31^ It is of interest to note that PANI:PSS globular shape revealed a smother morphology when compared with PPy:PSS, possibly originated by a coalescence phenomenon produced during the drying process.

These four aqueous suspensions were used as the basis for IJP ink formulations. The studied suspensions were formulated using a surfactant (1, 5% (w/v) of Triton X100) to adjust the surface tension of the ink prior to printing. Surface tension data of the evaluated formulation inks are detailed in Table S2 of the Supporting Information. Furthermore, to increase electric conductivity, dimethyl sulfoxide (DMSO) and glycerol (Gly) were used as a secondary dopant. Gly addition also contributes to adjust the viscosity of the CP formulation and the presence of DMSO and Gly shows a stabilization of the polymer benzoic structure once the film is formed.^32−37^ Hence, this intrinsic conjugation of the benzoic structure allows a better electron mobility through polymeric chains, which in turn enhances the electroactivity of CPs. This effect, which is broadly studied for polymers such as the gold standard PEDOT:PSS, is not so widely studied for PANI and PPy formulations and even less when they are applied as a pH sensor.

### Polymeric Ink Printing Process

Once formulated, the inks were printed using an inkjet printer to obtain a sensor array. Printing parameters were adjusted to obtain a continuous drop ejection for PANI:PSS(I) and (II) and PPy:PSS(I) and (II) ink printing. Waveform and printing parameters were obtained considering polymeric ink properties to have a reliable printing process of developed polymeric inks (Supporting Information Figure S7). As described in the literature, the ink–substrate interaction alters ink deposition, hence the drop spacing (DS) selection between two consecutive drops and the printer platen temperature determines the quality of the printed pattern on a specific substrate.^38^ The DS determines the resolution and density in *X*- and *Y*-axes of the printed samples; for example, a DS of 15 μm represents a print resolution of 1693 dpi (dots per inch). The platen temperature plays a critical role in drop evaporation that it is directly related to line formation, pattern definition, and coffee-ring effect. To evaluate an optimum DS, a line pattern test was performed with DS from 5 to 140 μm.^39^

**Figure 3:**
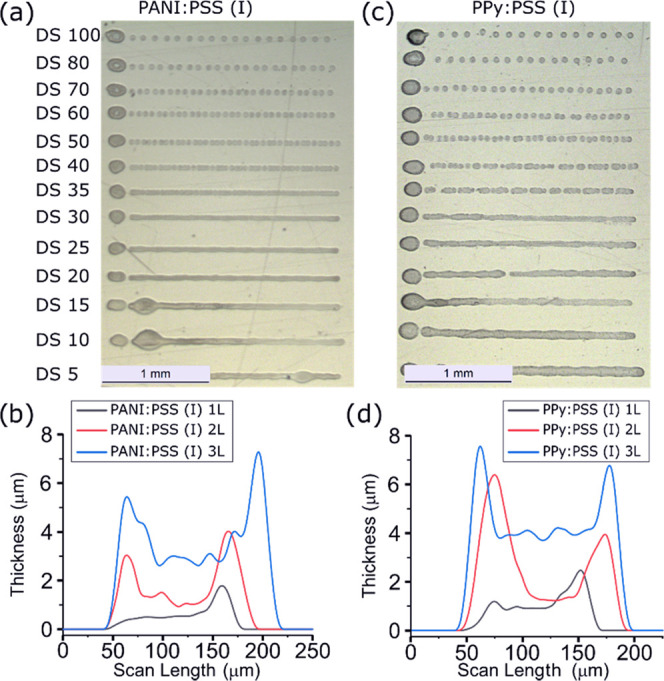
Line patterns printed on the PEN substrate for (a) PANI:PSS(I) and (b) PPy:PSS(I) formulated inks. Cross-sectional profile for (c) PANI:PSS(I) and (d) PPy:PSS(II) for 1L, 2L, or 3L.

Figure 3 shows the printed line patterns for PANI:PSS(I) (a) and PPy:PSS(I) (b). Both inks present more or less the same behavior and no main differences can be observed with the other formulations using *M*_w_ 200 000 PSS (data not shown). However, depending on the selected DS, different performance can be observed. When the chosen DS is too small (5 μm), bulging effects appear due to an excess of deposited material. For DS values of 40 μm or higher, the formation of isolated drops that are not able to form a continuous line can be observed. The proposed inks have optimized DS values between DS 10 and 35 μm that allow the printing of a homogeneous and continuous line. Apart from the line pattern evaluation, it has been observed experimentally that DS values higher than 10 μm do not allow the overlap of contiguous printed lines, as can be observed in Supporting Information Figure S8. This can be explained by a fast evaporation of ink solvents or interaction between the ink and substrate helped by drop kinetics. Also optimized was the substrate temperature, which was set to 40 °C to obtain a balance between solvent evaporation, ink spreading, and linewidth. Temperatures lower than 40 °C produced an excessive spreading and formation of inconsistent lines due to the slower evaporation of the ink, and temperatures higher than 40 °C showed thinner lines and exaggerated coffee-ring effects as a result of rapid solvent evaporation. Finally, a DS of 10 μm at 40 °C was determined as the optimal point to obtain the most homogeneous lines with a controlled coffee-ring effect to print the polymeric suspensions onto the Au microelectrodes.

Furthermore, different number of CP layers were printed wet-on-wet and evaluated later in the profilometer (one (1L), two (2L), and three layers (3L)). A desired thickness of around 1–3 μm should be achieved to decrease the CP film electrical resistance, decrease the pH behavior hysteresis, increase the pH sensor lifetime, and ensure a good repeatability as previously reported.^40−42^ Four layers (4L) were also tested; however, these conditions were not under consideration because the films were completely detached when being immersed in an aqueous solution, as shown in Figure S9, due to an excess of material over the metallic electrode. Profilometer measurements are shown in Figure 3c for PANI:PSS(I) and in Figure 3d for PPy:PSS(II); the well-known coffee-ring effect was observed for all of the printed layers, 3L being necessary for achieving a nonuniform thickness of around 3 μm in the central part of the pattern. After printing, PANI and PPy films with PSS of *M*_w_ 70 000 revealed a good stability in aqueous media, indicating its suitability as a sensor. However, when a PSS of *M*_w_ 200 000 was used, the polymeric film was completely detached after a few minutes of being in contact with the aqueous media. Thus, we selected the PANI:PSS(I) and PPy:PSS(I) inks for its evaluation and validation as a pH sensor.

### Evaluation of the Printed CP Inks as pH Sensors

For the evaluation of the polymeric printed films, potentiometric pH measurements were carried out by measuring the open-circuit potential at room temperature. Sensors were placed three times in the same buffer solution to evaluate the pH response and repeatability of the polymeric film. Considering the applicability of this sensor in biological applications, in addition to evaluating its sensitivity and linearity, special attention was paid to the physiological pH range (around pH 7.5).

**Figure 4:**
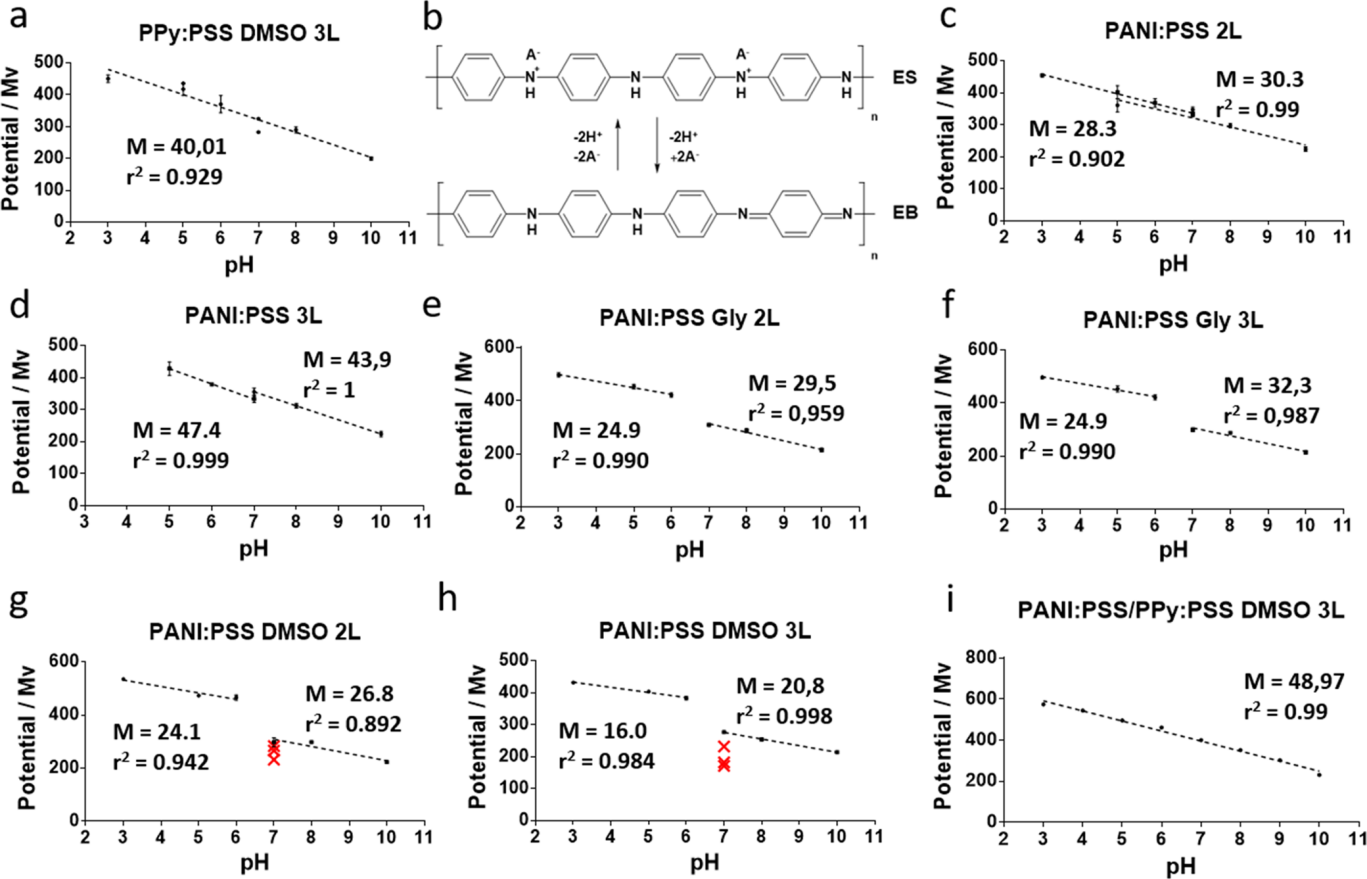
Plots of potentials against pH value reading from pH-meter for (a) PPy:PSS + DMSO 3L, (b) structure of PANI macromolecule changes due to protonation/deprotonation, (c) PANI:PSS 2L, (d) PANI:PSS 3L, (e) PANI:PSS + Gly 2L, (f) PANI:PSS + Gly 3L, (g) PANI:PSS + DMSO 2L, (h) PANI:PSS + DMSO 3L, and (i) PANI:PSS/PPy:PSS + DMSO 3L (*n* = 3, variation coefficient below 5%).

Figure 4 shows the potentiometric pH measurements of the evaluated formulations of PANI:PSS(I) and PPy:PSS(I). PPy:PSS(I) formulation revealed a poor linearity in the selected pH range even when a secondary dopant (DMSO) was used to enhance the polymer electroactivity (Figure 4a). This behavior of PPy as a pH sensor is described in the literature for similar devices.^43^

For PANI:PSS(I), the potential decreases with the increase of the pH, revealing two good linear regions similar to the behavior of electrodeposited PANI pH sensors but with less pronounced changes in the slopes.^44^ The presence of these regions is ascribed to the pH-dependent emeraldine salt (ES)–emeraldine base (EB) transition of PANI macromolecules, where, depending on the pH, there is a different level of protonation of the EB imine groups (Figure 4b). This protonation–deprotonation of PANI structure provides its electroactive properties allowing changes in the potential output signal, which reveals two different slopes corresponding to the protonation levels, where the acid pH region allows higher slope values, indicating better electron mobility and high electroactive behavior of the polymer. A result of this behavior can be seen in the printed sensors of PANI:PSS(I) without conductivity promoters, where two linear regions can be observed in Figure 4c,d, corresponding to a film of 2L or 3L. The linear regions present in PANI:PSS(I) 2L and 3L films presented similar slopes (28.3 mV/pH for the pH 3–7 region and 30.3 mV/pH for the pH 7–10 region). These changes may be attributed to the presence of sulfonate groups in PSS, which generates local acidic regions that allow the regulation of PANI protonation. Although PANI:PSS films reduce the effect of the change in the structure by improving the linearity of the sensor response, the neutral pH zone still presents a poorly defined signal. PANI:PSS(I) films formulated with a conductivity promoter such as glycerol (Figure 4e,f) or DMSO (Figure 4g,h) only magnified the effect of structure protonation, presenting higher differences between the potential output signals of the two regions. This effect is clearly visible for PANI:PSS samples with DMSO, where the measurement performed at pH 7 presented a high deviation. Increasing the number of layers of printed PANI:PSS film from 2 to 3 did not produce any significant improvement in this sense.

To improve the measurements in the physiological region as well as the linearity in the studied range of pH, we used a formulation that combines PANI:PSS(I) and PPy:PSS(I) (PANI:PSS/PPy:PSS). We hypothesized that when electron mobility cannot be carried out through the PANI chain due to the EB/ES structure change, the PPy chain supports electron mobility. First, we optimized the IJP conditions of the two polymers together. It was observed that mixing PANI:PSS(I) with PPy:PSS(I) (1:1 v/v) allowed the printability of a stable polymeric sensor. Figure S10 of the Supporting Information shows the line pattern of this new ink and the corresponding cross-sectional profile for 1L to 3L, as previously done for PANI and PPy base inks. In this case, the DS optimized is also 10 μm. However, the cross-sectional profiles show how the ink accumulates in the central part, noted by the distribution of the material. We observed that the most homogeneous film was achieved with 3L, with a thickness of about 5 μm.

Second, we studied the sensor response at different pH values. Figure 4i shows potential against pH plot for the PANI:PSS/PPy:PSS sensor. The potentiometric pH measurements revealed a good linearity in the range of pH 3–10. These results confirm our hypothesis because not only did we solve the drawbacks of PANI:PSS at the physiological range of pH but we also increased the stability of the PPy:PSS films. Furthermore, the sub-Nernstian response was in good agreement with other reported sensitivities for polymeric pH-based sensors.^18,44−47^ All measurements subsequently performed were done with the PANI:PSS/PPy:PSS ink printed over the Au microelectrodes with a platen temperature of 40 °C, DS of 10 μm, and 3L.

**Figure 5:**
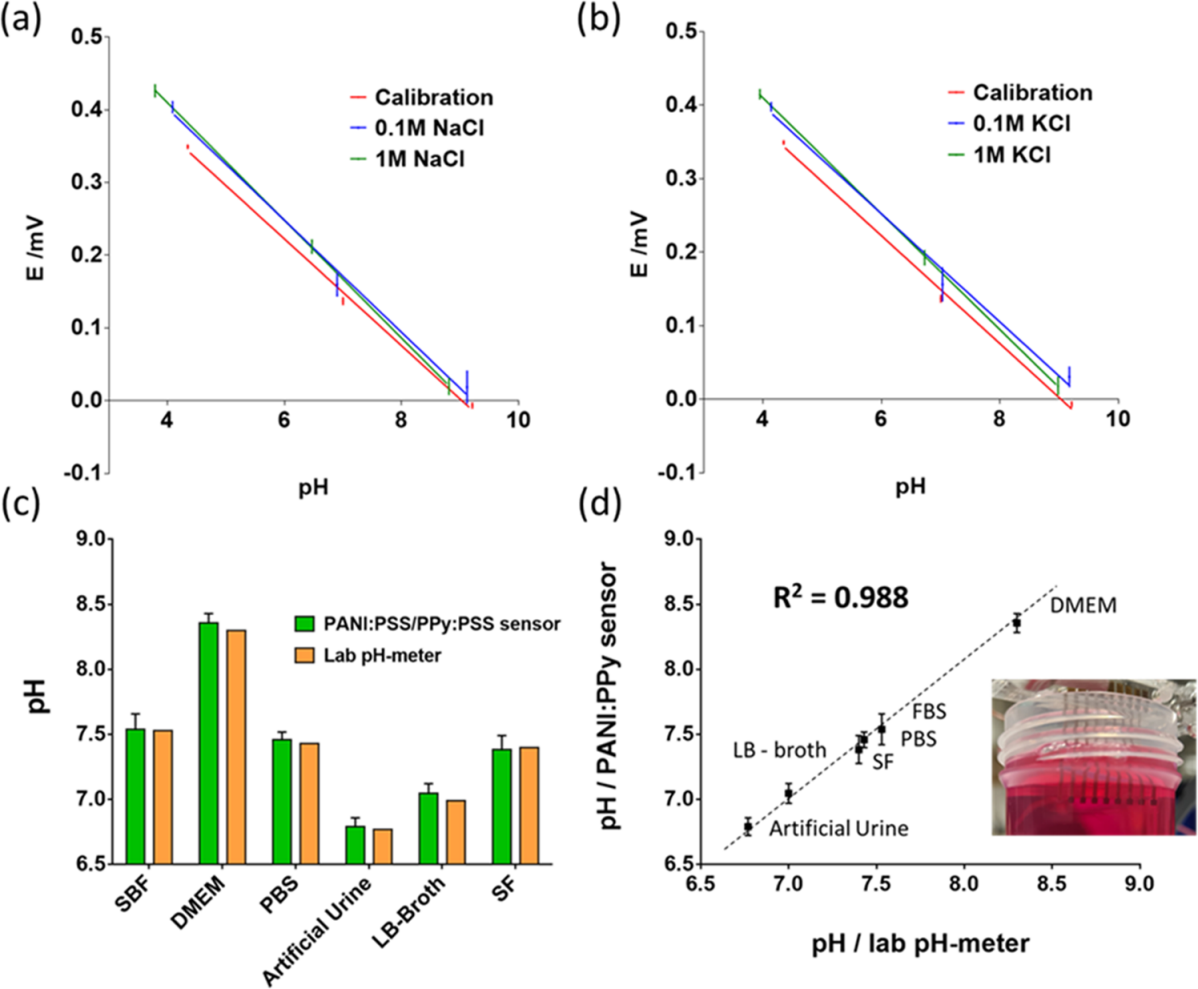
Potential interfering ion test for PANI:PSS/PPy:PSS sensors: (a) Na^+^ and (b) K^+^. Determination of pH value for a set of mediums used in the bioengineering field: (c) Comparison between PANI:PSS/PPy:PSS sensors (*n* = 3) with a laboratory pH-meter and (d) compared using a linear correlation.

Selectivity is one of the most important factors in potentiometric sensors and defines the ability to specifically measure hydronium ions in the presence of other ions. For this reason, we evaluated the potentiometric selectivity performance of the developed pH sensor against major potential interfering sodium (Na^+^) and potassium (K^+^) ions, observing the voltage drifts at different pH values for constant ion concentrations (Figure 5a,b). Potential against pH plots of buffered solution in the presence of Na^+^ and K^+^ revealed small voltage drifts similar to other similar devices described in the literature.^48,49^ Based on these results, the presence of Na^+^ and K^+^ does not interfere with the pH measurements of real unknown solutions. In this context, the performance of the PANI:PSS/PPy:PSS sensor was evaluated using six standard solutions of unknown pH used in the field of bioengineering: simulated body fluid (SBF), Dulbecco’s modified Eagle medium (DMEM), phosphate buffered saline (PBS), artificial urine, Luria-Bertani broth (LB-broth), and commercial Corning SF medium. Figure 5c shows the pH value of the solutions measured using the PANI:PSS/PPy:PSS sensor compared with a conventional pH-meter with a glass electrode. The pH values of these real solutions were determined revealing no significant differences between the measurements carried out with the PANI:PSS/PPy:PSS sensor and with the laboratory pH-meter. From a qualitative point of view, Figure 5d shows the correlation of the pH values of the two electrodes for the six evaluated solutions. As shown, the linear regression presents a coefficient of determination of 0.988, indicating the good agreement between values and demonstrating that the developed sensors present the same behavior as a laboratory device.

### Effect of the Gold Substrate on the Nernstian Behavior of the pH Sensor Response

In this section, we explore the utilization of an Au-based printed electrode modified with phthalocyanine (Pc) as a method to increase the sensitivity of the polymeric-based pH sensor. The commercial Au–Pc metallic ink used to print the Au electrodes was modified with a derivative metal-free phthalocyanine, which presented a delocalized planar structure, promoting the formation of large π-conjugated regions. These regions, directly in contact with the surface of the Au nanoparticle, improved the electrical pathway among them,^50^ allowing good conduction characteristics equivalent to those of the conventional nanoinks (Au-Np) with a minimum heat treatment (usually, sintering at 100–120 °C for several minutes gives well-conducted patterns). As previously described, the incorporation of phthalocyanine into PPy or PANI chains results in the increase of the delocalized regions, enhancing the doping level of the CP, the electron mobility, and the electroactivity.^51,52^

**Figure 6:**
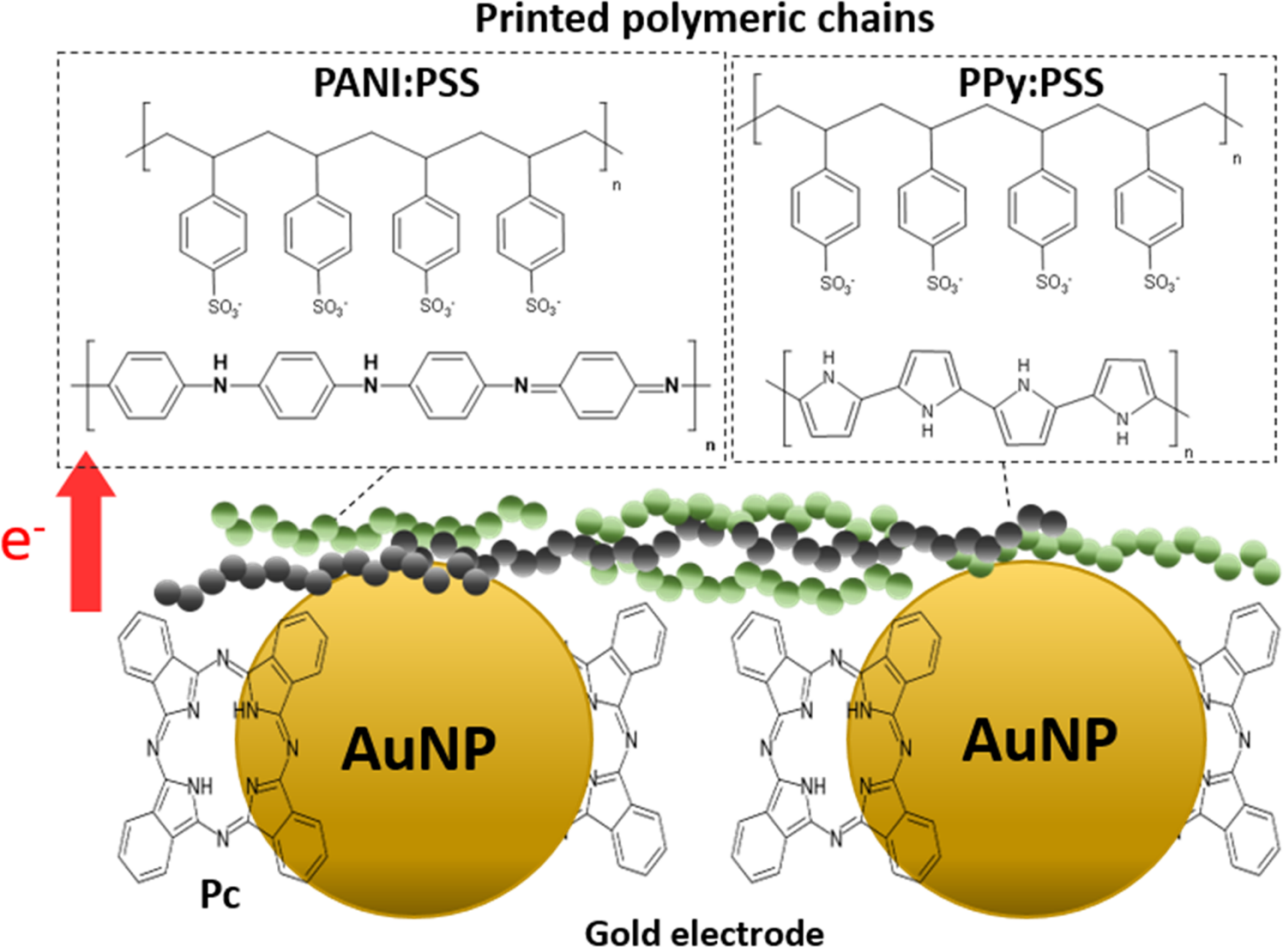
Schematic illustration of π-junction of Au–Pc ink modified with phthalocyanine and PANI:PSS/PPy:PSS polymeric chain.

Figure 6 shows the interactions of the Au–Pc ink together with the CPs. Once the PANI:PSS/PPy:PSS ink was printed onto the Au–Pc microelectrode, the π-conjugated regions of the PANI, PPy, and PSS interacted with the π-conjugated regions of Au–Pc, enhancing the electron mobility between the printed Au–Pc substrate and the polymeric film in comparison with the bare Au-Np ink.

**Figure 7:**
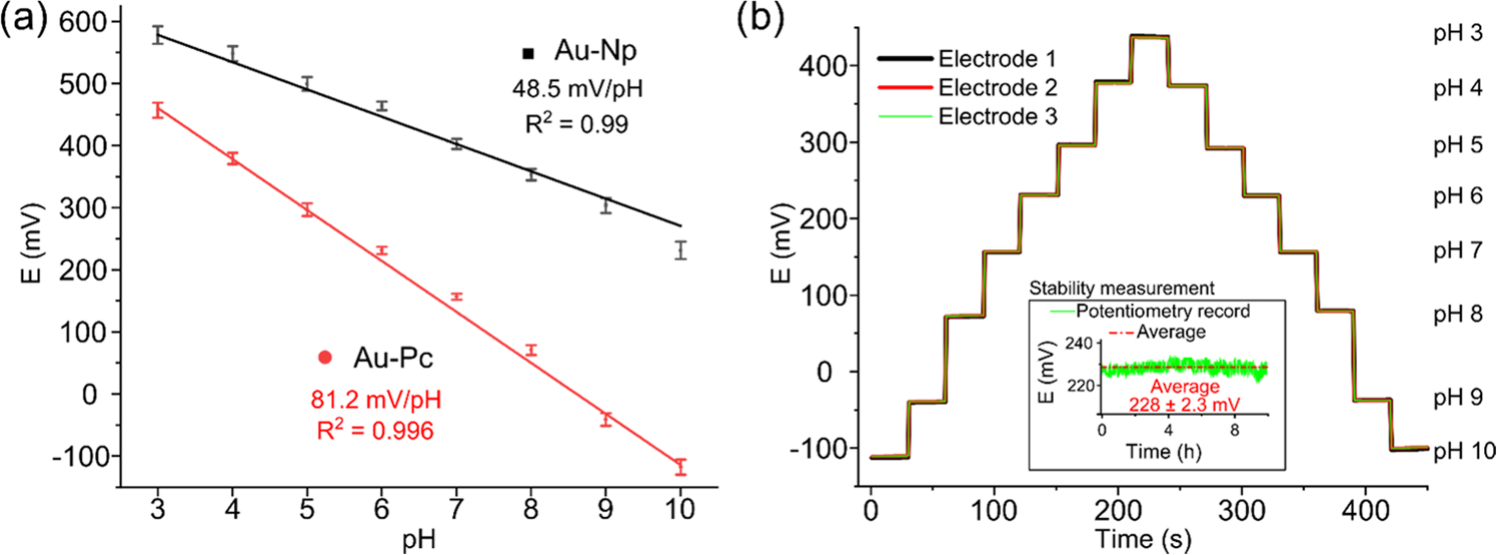
Calibration curves of PANI:PSS/PPy:PSS. (a) Sensor sensitivity of PANI:PSS/PPy:PSS on Au-Np and Au–Pc microelectrodes in the pH range of 3–10 (*n* = 3, variation coefficient below 3% for Au–Pc and below 4% for Au-Np). (b) Repeatability of the electrode of PANI:PSS/PPy:PSS on Au–Pc at different pH values from 3 to 10 (Inset: midterm pH sensor stability at pH 6 for 10 h).

Figure 7a shows the effect of Pc in direct contact with the PANI:PSS/PPy:PSS-based sensor. pH calibration was performed for PANI:PSS/PPy:PSS printed on the nonmodified Au substrate (Au-Np) revealing a Nernstian sensitivity of 48.5 ± 0.5 mV/pH with a high correlation coefficient *r*^2^ of 0.990 in all of the pH ranges (3–10), in good agreement with other previously reported CP-based pH sensors.^41^ When the PANI:PSS/PPy:PSS polymeric film was printed onto the Au–Pc, a super-Nernstian response of 81.2 ± 0.5 mV/pH with high linearity was achieved. Table 1 summarizes the most recent papers on conducting polymers used for pH sensing. As can be observed, our work has the highest sensitivities achieved for a polymeric-based sensor.^17^ As stated before, this increase in sensitivity can be explained due to enhanced electron mobility between the Au–Pc substrate and PANI:PSS/PPy:PSS. The utilization of Au–Pc ink to generate a high delocalized π-region between the electrode and the PANI:PSS/PPy:PSS printed polymeric film is not only interesting in terms of obtaining a highly sensitive pH sensor but it also describes a strategy that could be implemented in other CPs to increase electroactivity, thus unlocking new applications.

**Table 1:** Comparison of Selected Works from the Literature, Highlighting the Conducting Polymers Used for pH Sensing, the Fabrication Technique Used for Deposition of the Conducting Polymer, pH Range, and Sensitivity^a^

conducting polymer	technique	pH range	sensitivity (mV/pH)	refs
PPy	electropolymerization	2–12	54.67 ± 0.7	(53)
PANI nanofibers	polymerization	3.9–10.1	62.4	(54)
PANI nanopillar	soft lithography	2.38–11.61	60.3	(55)
PANI	electropolymerization	5–7		(21)
PANI	electropolymerization	5.5–8	59.2	(22)
PPy + CNT, PANI + CNT	electrodeposition	1–13	59	(56)
PANI	laser carbonized	4–10	51	(57)
PANI	coating	4–10	50	(41)
PANI	coating	4–8	54 ± 0.51	(58)
PANI + CNT	electrodeposition	1–13	58	(59)
PPy	electropolymerization	3–10	46	(60)
PANI + DBSA	spin coating	5.4–8.6	58.57	(61)
PANI + MWCNT	screen printing	2–11	20.63	(62)
PANI + PU	electrospinning	2– 7	60	(63)
PANI	electrodeposition	4–8	60.6	(64)
PANI + PPy + PSS	inkjet printing	3–10	81.2 ± 0.5	this work

a Abbreviations: CNT, carbon nanotubes; MWCNT, multiwalled carbon nanotubes; DBSA, dodecyl benzene sulfonic acid; PU, polyurethane; PSS, sodium 4-styrenesulfonate.

The complete evaluation of the pH-sensing characteristics required a reproducibility study of the PANI:PSS/PPy:PSS printed on top of the Au–Pc microelectrode. Three independent microelectrodes, made from the same fabrication batch, were measured by OCP and the base-to-acid and acid-to-base changes were studied. Figure 7b shows the response of each individual microelectrode, and as can be observed, the potential responses for each one almost overlap, indicating an excellent reproducibility. The average sensitivity of the three electrodes is 81.2 mV/pH with an average variation coefficient for each pH level under 0.5% (Table S3), confirming a good repeatability of PANI:PSS/PPy:PSS sensor with a correlation coefficient larger than 0.996 for all measurements. The midterm stability of the pH sensor near protonation and deprotonation regions was further investigated by measuring its potential drift over 10 h at room temperature. For this purpose, the platform was immersed in pH 6 buffered solution (Figure 7b inset) and continuously measured. Although some slight potential fluctuations can be explained by temperature changes, the sensor shows an excellent stability of continuous reading over a 10 h test resulting in a potential drift of 0.2 mV/h, an average reading of 228 mV, and a standard deviation of 2.3 mV, thus providing reliable pH read-outs for long measurements.

### Validation of the PANI:PSS/PPy:PSS Film as pH Sensor

**Figure 8:**
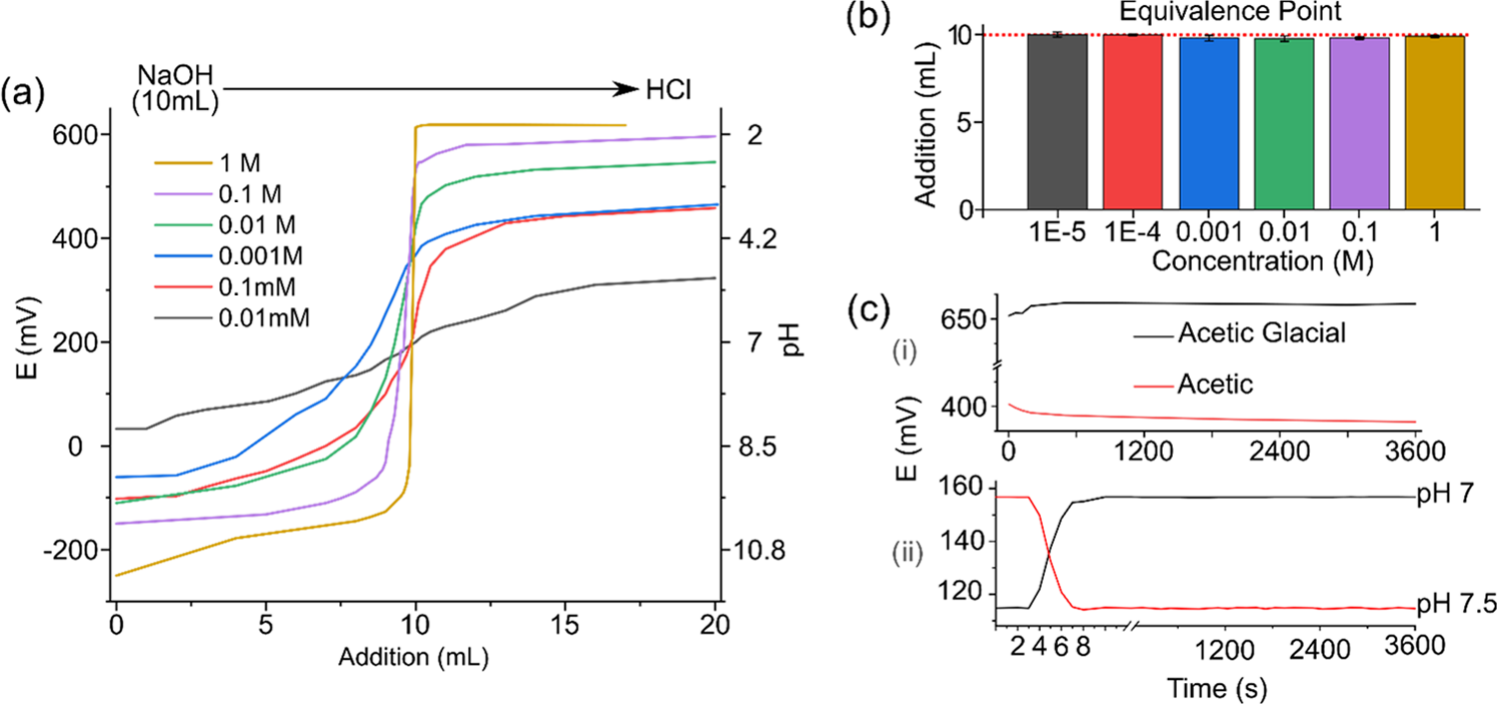
Titration of strong base with strong acid. (a) Potentiometric pH titration curves for NaOH and HCl at the indicated molarity, (b) representation of the equivalence point for each NaOHmolarity. The red dotted line represents the theoretical values, and (c) stability of the pH sensor in acid conditions, acetic, and acetic glacial acid (i) and buffer solutions at pH 7 and 7.5 (ii).

To evaluate if the PANI:PSS/PPy:PSS sensor is a good candidate for local pH determination, pH titration was studied. Titration was performed over a range of molarities (from 0.01 mM to 1 M) from NaOH to HCl by addition of a specific volume of acid. Figure 8a shows the titration plot of aqueous NaOH with HCl. Results obtained for monoprotic titration of strong alkalies with strong acids are comparable with results obtained with a commercial glass electrode. The PANI:PSS/PPy:PSS sensor presented had a reasonable detection limit (0.01 mM), a concentration range (from 1 M to 0.01 mM), and a pH range (from 3 to 10) compared with previous studies.^17,65^ The theoretical equivalence point for this titration is 10 mL at pH 7, due to p*K*_a_ of NaOH and HCl. Figure 8b shows the experimental calculated equivalence points for all concentrations and their standard deviation. The sensors present good performance and accuracy, allowing detection of pH changes even ±0.01 pH units with a high sensitivity of 81.05 ± 0.08 mV/pH. The same behavior was observed when the titration was performed from the acidic to basic range as shown in Figure S11. An equivalence point determined at pH 7 of 10 mL and a detection limit of 0.01 mM was obtained.

Figure 8c-i shows the stability of PANI:PSS/PPy:PSS sensor for 1 h in acetic acid and acetic glacial acid showing a stabilization time of 300 s for these low pH values, a small drift of 1 mV/h for acetic glacial, and 6 mV/h for acetic acid. This proves the good behavior of the sensor in extreme pH conditions, allowing its utilization on nonaqueous media. The stability was also measured in aqueous buffer solutions at pH 7 and 7.5 (Figure 8c-ii) to demonstrate how the sensors met the proposed benchmarks, showing that the response time was achieved within 7 s and a small drift of 1.5 mV/h for pH 7 and 1.8 mV/h for pH 7.5. The sensitivity did not alter after 2 h in extreme conditions, obtaining values of 80.7 mV/pH of the PANI:PSS/PPy:PSS sensor (data not shown). This result demonstrates that PANI:PSS/PPy:PSS polymeric film is not affected after performing measurements in nonaqueous acid media. Additionally, since the substrate proposed is flexible, the pH sensor evaluation was completed, verifying that the sensor response remained unaltered after repeated bending tests. For this, a proof of concept was carried out to verify how sensor sensitivity evolves after being subjected to different bending cycles (Figure S12 of the Supporting Information). It was found that after 50 bending cycles of 90°, the sensitivity of the sensors remained stable around 77 and 78 mV/pH, demonstrating that the sensor flexibility did not affect the pH sensor response.

## Conclusions

We have presented a novel approach for fabricating a stable pH sensor using a highly rough printed Au ink as a substrate metal electrode. The pH sensors were completely fabricated with IJP based on PANI:PSS/PPy:PSS inks deposited on a gold microelectrode printed on a flexible substrate. The different approaches allowed the optimization of pH sensor properties and, in particular, IJP ink formulation allowed the improvement of the pH sensor sensitivity. Furthermore, the combination of PANI:PSS and PPy:PSS particles improved the linearity of pH against potential as well as expanding the pH range (from pH 3 to 10) and resolving problems of PANI sensors regarding physiological pH, thereby promoting utilization in biological applications. The use of a gold ink modified with Pc for the gold microelectrode substrate revealed an enhancement of electron mobility between the conducting polymer chains and the gold nanoparticles, generating a delocalized π-region that improved the electroactivity. In this regard, the sensor reached a linear super-Nernstian response (81.2 ± 0.5 mV/pH unit), one of the highest sensitivity values for a polymeric pH sensor reported to date.

The characterization of the sensor also revealed a wide range of versatile properties that extends the range of possible applications. Furthermore, the sensor presented high stability in aqueous and nonaqueous media (acetic acid), confirming its ability to perform highly accurate titration measurements by detecting small changes in the concentration of strong monoprotic alkalies with strong acid (0.01 M).

## Supporting Information Available

Pseudoreference electrode chlorination and stability characterization; ink formulations; surface tension ink formulations; IR spectra characterization; CP suspensions—pH dependence; DLS characterization; ζ-potential characterization; FESEM images; parameters of the printing process; images of printing test and nonadherence of polymeric film; PANI:PSS/PPy:PSS line pattern study; variation coefficient; titration of strong acid with strong base; and pH sensor sensitivity after bending (PDF)

## References

[ref1] Bandodkar A. J.; Jeerapan I.; Wang J. Wearable Chemical Sensors: Present Challenges and Future Prospects. ACS Sens. 2016, 1, 464–482. 10.1021/acssensors.6b00250.

[ref2] Jin W.; Wu L.; Song Y.; Jiang J.; Zhu X.; Yang D.; Bai C. Continuous Intra-Arterial Blood PH Monitoring by a Fiber-Optic Fluorosensor. IEEE Trans. Biomed. Eng. 2011, 58, 1232–1238. 10.1109/TBME.2011.2107514. 21257367

[ref3] Chaisiwamongkhol K.; Batchelor-McAuley C.; Compton R. G. Optimising Amperometric PH Sensing in Blood Samples: An Iridium Oxide Electrode for Blood PH Sensing. Analyst 2019, 144, 1386–1393. 10.1039/C8AN02238K. 30569049

[ref4] Dang W.; Manjakkal L.; Navaraj W. T.; Lorenzelli L.; Vinciguerra V.; Dahiya R. Stretchable Wireless System for Sweat PH Monitoring. Biosens. Bioelectron. 2018, 107, 192–202. 10.1016/j.bios.2018.02.025. 29471280

[ref5] Nakata S.; Shiomi M.; Fujita Y.; Arie T.; Akita S.; Takei K. A Wearable PH Sensor with High Sensitivity Based on a Flexible Charge-Coupled Device. Nat. Electron. 2018, 1, 596–603. 10.1038/s41928-018-0162-5.

[ref6] Zhao P.; Tang Z.; Chen X.; He Z.; He X.; Zhang M.; Liu Y.; Ren D.; Zhao K.; Bu W. Ferrous-Cysteine–Phosphotungstate Nanoagent with Neutral PH Fenton Reaction Activity for Enhanced Cancer Chemodynamic Therapy. Mater. Horiz. 2019, 6, 369–374. 10.1039/C8MH01176A.

[ref7] Ray T. R.; Choi J.; Bandodkar A. J.; Krishnan S.; Gutruf P.; Tian L.; Ghaffari R.; Rogers J. A. Bio-Integrated Wearable Systems: A Comprehensive Review. Chem. Rev. 2019, 119, 5461–5533. 10.1021/acs.chemrev.8b00573. 30689360

[ref8] Flexible Electronics Market Size Growth | Industry Forecast Report 2024. https://www.grandviewresearch.com/industry-analysis/flexible-electronics-market/toc (accessed Jun 4 2020).

[ref9] Yin Z.; Huang Y.; Bu N.; Wang X.; Xiong Y. Inkjet Printing for Flexible Electronics: Materials, Processes and Equipments. Chin. Sci. Bull. 2010, 55, 3383–3407. 10.1007/s11434-010-3251-y.

[ref10] Sui Y.; Zorman C. A. Review—Inkjet Printing of Metal Structures for Electrochemical Sensor Applications. J. Electrochem. Soc. 2020, 167, 037571 10.1149/1945-7111/ab721f.

[ref11] Sundriyal P.; Bhattacharya S. Inkjet-Printed Sensors on Flexible Substrates. In Energy, Environment, and Sustainability, Bhattacharya S.; Agarwal A. K.; Chanda N.; Pandey A.; Sen A. K., Eds.; Springer: Singapore, 2018; pp 89–113.

[ref12] Qin Y.; Kwon H.-J.; Howlader M. M. R.; Deen M. J. Microfabricated Electrochemical PH and Free Chlorine Sensors for Water Quality Monitoring: Recent Advances and Research Challenges. RSC Adv. 2015, 5, 69086–69109. 10.1039/C5RA11291E.

[ref13] Zea M.; Moya A.; Fritsch M.; Ramon E.; Villa R.; Gabriel G. Enhanced Performance Stability of Iridium Oxide Based PH Sensors Fabricated on Rough Inkjet-Printed Platinum. ACS Appl. Mater. Interfaces 2019, 11, 15160–15169. 10.1021/acsami.9b03085. 30848584

[ref14] Xu Z.; Dong Q.; Otieno B.; Liu Y.; Williams I.; Cai D.; Li Y.; Lei Y.; Li B. Real-Time in Situ Sensing of Multiple Water Quality Related Parameters Using Micro-Electrode Array (MEA) Fabricated by Inkjet-Printing Technology (IPT). Sens. Actuators, B 2016, 237, 1108–1119. 10.1016/j.snb.2016.09.040.

[ref15] Qin Y.; Alam A. U.; Howlader M. M. R.; Hu N.-X.; Deen M. J. Inkjet Printing of a Highly Loaded Palladium Ink for Integrated, Low-Cost PH Sensors. Adv. Funct. Mater. 2016, 26, 4923–4933. 10.1002/adfm.201600657.

[ref16] Määttänen A.; Vanamo U.; Ihalainen P.; Pulkkinen P.; Tenhu H.; Bobacka J.; Peltonen J. A Low-Cost Paper-Based Inkjet-Printed Platform for Electrochemical Analyses. Sens. Actuators, B 2013, 177, 153–162. 10.1016/j.snb.2012.10.113.

[ref17] Alam A. U.; Qin Y.; Nambiar S.; Yeow J. T. W.; Howlader M. M. R.; Hu N. X.; Deen M. J. Polymers and Organic Materials-Based PH Sensors for Healthcare Applications. Prog. Mater. Sci. 2018, 96, 174–216. 10.1016/j.pmatsci.2018.03.008.

[ref18] Korostynska O.; Arshak K.; Gill E.; Arshak A. Review on State-of-the-Art in Polymer Based PH Sensors. Sensors 2007, 7, 3027–3042. 10.3390/s7123027. 28903277 PMC3841878

[ref19] Binag C. A.; Bartolome A. J.; Tongol B.; Santiago K. S. Electronically Synthesized Polymer-Based PH Sensors. Philipp. J. Sci. 1999, 128, 247–252.

[ref20] Kocak G.; Tuncer C.; Bütün V. PH-Responsive Polymers. Polym. Chem. 2017, 8, 144–176. 10.1039/C6PY01872F.

[ref21] Fanzio P.; Chang C.-T.; Skolimowski M.; Tanzi S.; Sasso L. Fully-Polymeric PH Sensor Realized by Means of a Single-Step Soft Embossing Technique. Sensors 2017, 17, 1169 10.3390/s17051169. PMC5470914 28531106

[ref22] Guinovart T.; Valdés-Ramírez G.; Windmiller J. R.; Andrade F. J.; Wang J. Bandage-Based Wearable Potentiometric Sensor for Monitoring Wound PH. Electroanalysis 2014, 26, 1345–1353. 10.1002/elan.201300558.

[ref23] Zea M.; Moya A.; Abrao-Nemeir I.; Gallardo-Gonzalez J.; Zine N.; Errachid A.; Villa R.; Gabriel G. In All Inkjet Printing Sensor Device on Paper: For Immunosensors Applications, 2019 20th International Conference on Solid-State Sensors, Actuators and Microsystems Eurosensors XXXIII (TRANSDUCERS EUROSENSORS XXXIII), 2019; pp 2472–2475.

[ref24] Park Y. R.; Doh J. H.; Shin K.; Seo Y. S.; Kim Y. S.; Kim S. Y.; Choi W. K.; Hong Y. J. Solution-Processed Quantum Dot Light-Emitting Diodes with PANI:PSS Hole-Transport Interlayers. Org. Electron. 2015, 19, 131–139. 10.1016/j.orgel.2014.12.030.

[ref25] Jang J.; Ha J.; Cho J. Fabrication of Water-Dispersible Polyaniline-Poly(4-Styrenesulfonate) Nanoparticles For Inkjet-Printed Chemical-Sensor Applications. Adv. Mater. 2007, 19, 1772–1775. 10.1002/adma.200602127.

[ref26] Han H.; Lee J. S.; Cho S. Comparative Studies on Two-Electrode Symmetric Supercapacitors Based on Polypyrrole: Poly(4- Styrenesulfonate) with Different Molecular Weights of Poly(4-Styrenesulfonate). Polymers 2019, 11, 232 10.3390/polym11020232. PMC6419067 30960216

[ref27] Texidó R.; Borrós S. Allylamine PECVD Modification of PDMS as Simple Method to Obtain Conductive Flexible Polypyrrole Thin Films. Polymers 2019, 11, 2108 10.3390/polym11122108. PMC6960888 31847507

[ref28] Texidó R.; Orgaz A.; Ramos V.; Borrós S. Stretchable Conductive Polypyrrole Films Modified with Dopaminated Hyaluronic Acid. Mater. Sci. Eng., C 2017, 76, 295–300. 10.1016/j.msec.2017.03.072. 28482530

[ref29] Nardes A. M.; Kemerink M.; Janssen R. A. J.; Bastiaansen J. A. M.; Kiggen N. M. M.; Langeveld B. M. W.; Van Breemen A. J. J. M.; De Kok M. M. Microscopic Understanding of the Anisotropic Conductivity of PEDOT:PSS Thin Films. Adv. Mater. 2007, 19, 1196–1200. 10.1002/adma.200602575.

[ref30] Lang U.; Muller E.; Naujoks N.; Dual J. Microscopical Investigations of PEDOT:PSS Thin Films. Adv. Funct. Mater. 2009, 19, 1215–1220. 10.1002/adfm.200801258.

[ref31] Yan W.; Han J. Synthesis and Formation Mechanism Study of Rectangular-Sectioned Polypyrrole Micro/Nanotubules. Polymer 2007, 48, 6782–6790. 10.1016/j.polymer.2007.09.026.

[ref32] Zhang J.; Gao L.; Sun J.; Liu Y.; Wang Y.; Wang J. Incorporation of Single-Walled Carbon Nanotubes with PEDOT/PSS in DMSO for the Production of Transparent Conducting Films. Diamond Relat. Mater. 2012, 22, 82–87. 10.1016/j.diamond.2011.12.008.

[ref33] Eom S. H.; Senthilarasu S.; Uthirakumar P.; Yoon S. C.; Lim J.; Lee C.; Lim H. S.; Lee J.; Lee S. H. Polymer Solar Cells Based on Inkjet-Printed PEDOT:PSS Layer. Org. Electron. 2009, 10, 536–542. 10.1016/j.orgel.2009.01.015.

[ref34] Savagatrup S.; Chan E.; Renteria-Garcia S. M.; Printz A. D.; Zaretski A. V.; O’Connor T. F.; Rodriquez D.; Valle E.; Lipomi D. J. Plasticization of PEDOT:PSS by Common Additives for Mechanically Robust Organic Solar Cells and Wearable Sensors. Adv. Funct. Mater. 2015, 25, 427–436. 10.1002/adfm.201401758.

[ref35] Latonen R. M.; Määttänen A.; Ihalainen P.; Xu W.; Pesonen M.; Nurmi M.; Xu C. Conducting Ink Based on Cellulose Nanocrystals and Polyaniline for Flexographical Printing. J. Mater. Chem. C 2017, 5, 12172–12181. 10.1039/C7TC03729E.

[ref36] Ghamouss F.; Brugère A.; Anbalagan A. C.; Schmaltz B.; Luais E.; Tran-Van F. Novel Glycerol Assisted Synthesis of Polypyrrole Nanospheres and Its Electrochemical Properties. Synth. Met. 2013, 168, 9–15. 10.1016/j.synthmet.2013.02.005.

[ref37] Lee M. W.; Lee M. Y.; Choi J. C.; Park J. S.; Song C. K. Fine Patterning of Glycerol-Doped PEDOT:PSS on Hydrophobic PVP Dielectric with Ink Jet for Source and Drain Electrode of OTFTs. Org. Electron. 2010, 11, 854–859. 10.1016/j.orgel.2010.01.028.

[ref38] Oh Y.; Kim J.; Yoon Y. J.; Kim H.; Yoon H. G.; Lee S.-N.; Kim J. Inkjet Printing of Al2O3 Dots, Lines, and Films: From Uniform Dots to Uniform Films. Curr. Appl. Phys. 2011, 11, S359–S363. 10.1016/j.cap.2010.11.065.

[ref39] Moya A.; Sowade E.; del Campo F. J.; Mitra K. Y.; Ramon E.; Villa R.; Baumann R. R.; Gabriel G. All-Inkjet-Printed Dissolved Oxygen Sensors on Flexible Plastic Substrates. Org. Electron. 2016, 39, 168–176. 10.1016/j.orgel.2016.10.002.

[ref40] Lopez Aldaba A.; González-Vila Á.; Debliquy M.; Lopez-Amo M.; Caucheteur C.; Lahem D. Polyaniline-Coated Tilted Fiber Bragg Gratings for PH Sensing. Sens. Actuators, B 2018, 254, 1087–1093. 10.1016/j.snb.2017.07.167.

[ref41] Rahimi R.; Ochoa M.; Parupudi T.; Zhao X.; Yazdi I. K.; Dokmeci M. R.; Tamayol A.; Khademhosseini A.; Ziaie B. A Low-Cost Flexible PH Sensor Array for Wound Assessment. Sens. Actuators, B 2016, 229, 609–617. 10.1016/j.snb.2015.12.082.

[ref42] Lindfors T.; Ivaska A. PH Sensitivity of Polyaniline and Its Substituted Derivatives. J. Electroanal. Chem. 2002, 531, 43–52. 10.1016/S0022-0728(02)01005-7.

[ref43] Lakard B.; Segut O.; Lakard S.; Herlem G.; Gharbi T. Potentiometric Miniaturized PH Sensors Based on Polypyrrole Films. Sens. Actuators, B 2007, 122, 101–108. 10.1016/j.snb.2006.04.112.

[ref44] Gao W.; Song J. Polyaniline Film Based Amperometric PH Sensor Using a Novel Electrochemical Measurement System. Electroanalysis 2009, 21, 973–978. 10.1002/elan.200804500.

[ref45] Lakard B.; Segut O.; Lakard S.; Herlem G.; Gharbi T. Potentiometric Miniaturized PH Sensors Based on Polypyrrole Films. Sens. Actuators, B 2007, 122, 101–108. 10.1016/j.snb.2006.04.112.

[ref46] Lindfors T.; Ivaska A. PH Sensitivity of Polyaniline and Its Substituted Derivatives. J. Electroanal. Chem. 2002, 531, 43–52. 10.1016/S0022-0728(02)01005-7.

[ref47] Lakard B.; Segut O.; Lakard S.; Herlem G.; Gharbi T. Potentiometric Miniaturized PH Sensors Based on Polypyrrole Films. Sens. Actuators, B 2007, 122, 101–108. 10.1016/j.snb.2006.04.112.

[ref48] Masalles C.; Borrós S.; Viñas C.; Teixidor F. Simple PVC-PPy Electrode for PH Measurement and Titrations. Anal. Bioanal. Chem. 2002, 372, 513–518. 10.1007/s00216-001-1221-7. 11939624

[ref49] Dang W.; Manjakkal L.; Navaraj W. T.; Lorenzelli L.; Vinciguerra V.; Dahiya R. Stretchable Wireless System for Sweat PH Monitoring. Biosens. Bioelectron. 2018, 107, 192–202. 10.1016/j.bios.2018.02.025. 29471280

[ref50] Minari T.; Kanehara Y.; Liu C.; Sakamoto K.; Yasuda T.; Yaguchi A.; Tsukada S.; Kashizaki K.; Kanehara M. Room-Temperature Printing of Organic Thin-Film Transistors with π-Junction Gold Nanoparticles. Adv. Funct. Mater. 2014, 24, 4886–4892. 10.1002/adfm.201400169.

[ref51] Radhakrishnan S.; Deshpande S. D. Electrical Properties of Conducting Polypyrrole Films Functionalized with Phthalocyanine. Mater. Lett. 2001, 48, 144–150. 10.1016/S0167-577X(00)00294-9.

[ref52] Panwar V.; Kumar P.; Ray S. S.; Jain S. L. Organic Inorganic Hybrid Cobalt Phthalocyanine/Polyaniline as Efficient Catalyst for Aerobic Oxidation of Alcohols in Liquid Phase. Tetrahedron Lett. 2015, 56, 3948–3953. 10.1016/j.tetlet.2015.05.003.

[ref53] Prissanaroon-Ouajai W.; Pigram P. J.; Jones R.; Sirivat A. A Sensitive and Highly Stable Polypyrrole-Based PH Sensor with Hydroquinone Monosulfonate and Oxalate Co-Doping. Sens. Actuators, B 2009, 138, 504–511. 10.1016/j.snb.2009.01.037.

[ref54] Park H. J.; Yoon J. H.; Lee K. G.; Choi B. G. Potentiometric Performance of Flexible PH Sensor Based on Polyaniline Nanofiber Arrays. Nano Convergence 2019, 6, 9 10.1186/s40580-019-0179-0. 30880366 PMC6421353

[ref55] Yoon J. H.; Hong S. B.; Yun S.-O.; Lee S. J.; Lee T. J.; Lee K. G.; Choi B. G. High Performance Flexible PH Sensor Based on Polyaniline Nanopillar Array Electrode. J. Colloid Interface Sci. 2017, 490, 53–58. 10.1016/j.jcis.2016.11.033. 27870959

[ref56] Ferrer-Anglada N.; Kaempgen M.; Roth S. Transparent and Flexible Carbon Nanotube/Polypyrrole and Carbon Nanotube/Polyaniline PH Sensors. Phys. Status Solidi B 2006, 243, 3519–3523. 10.1002/pssb.200669220.

[ref57] Rahimi R.; Ochoa M.; Yu W.; Ziaie B. In A Highly Stretchable PH Sensor Array Using Elastomer-Embedded Laser Carbonized Patterns, 2015 Transducers - 2015 18th International Conference on Solid-State Sensors, Actuators and Microsystems (TRANSDUCERS), 2015; pp 1897–1900.

[ref58] Punjiya M.; Rezaei H.; Zeeshan M. A.; Sonkusale S. In A Flexible PH Sensing Smart Bandage with Wireless CMOS Readout for Chronic Wound Monitoring, 2017 19th International Conference on Solid-State Sensors, Actuators and Microsystems (TRANSDUCERS), 2017; pp 1700–1702.

[ref59] Kaempgen M.; Roth S. Transparent and Flexible Carbon Nanotube/Polyaniline PH Sensors. J. Electroanal. Chem. 2006, 586, 72–76. 10.1016/j.jelechem.2005.09.009.

[ref60] Aquino-Binag C. N.; Kumar N.; Lamb R. N.; Pigram P. J. Fabrication and Characterization of a Hydroquinone-Functionalized Polypyrrole Thin-Film PH Sensor. Chem. Mater. 1996, 8, 2579–2585. 10.1021/cm9506114.

[ref61] Li Y.; Mao Y.; Xiao C.; Xu X.; Li X. Flexible PH Sensor Based on a Conductive PANI Membrane for PH Monitoring. RSC Adv. 2020, 10, 21–28. 10.1039/C9RA09188B. PMC9047031 35492551

[ref62] Bao Q.; Yang Z.; Song Y.; Fan M.; Pan P.; Liu J.; Liao Z.; Wei J. Printed Flexible Bifunctional Electrochemical Urea-PH Sensor Based on Multiwalled Carbon Nanotube/Polyaniline Electronic Ink. J. Mater. Sci.: Mater. Electron. 2019, 30, 1751–1759. 10.1007/s10854-019-00923-y.

[ref63] Hou X.; Zhou Y.; Liu Y.; Wang L.; Wang J. Coaxial Electrospun Flexible PANI//PU Fibers as Highly Sensitive PH Wearable Sensor. J. Mater. Sci. 2020, 55, 16033–16047. 10.1007/s10853-020-05110-7.

[ref64] Wang R.; Zhai Q.; Zhao Y.; An T.; Gong S.; Guo Z.; Shi Q.; Yong Z.; Cheng W. Stretchable Gold Fiber-Based Wearable Electrochemical Sensor toward PH Monitoring. J. Mater. Chem. B 2020, 8, 3655–3660. 10.1039/C9TB02477H. 31998927

[ref65] Yu K.; He N.; Kumar N.; Wang N.; Bobacka J.; Ivaska A. Electrosynthesized Polypyrrole/Zeolite Composites as Solid Contact in Potassium Ion-Selective Electrode. Electrochim. Acta 2017, 228, 66–75. 10.1016/j.electacta.2017.01.009.

