# Supplementary material for: Specially Designed Polyaniline/Polypyrrole Ink for a Fully Printed Highly Sensitive pH Microsensor
Source: ACS Appl Mater Interfaces. 2021 Jul 6;13(28):33524–35. doi: 10.1021/acsami.1c08043 (PMC8397255; doi:10.1021/acsami.1c08043)
Supplement: Supplementary file 1 — am1c08043_si_001.pdf [file am1c08043_si_001.pdf]

Specially designed polyaniline/polypyrrole ink for a fully printed high sensitive pH microsensor.

Miguel Zea,†,‡, Robert Texido§, Rosa Villa†,¶, Salvador Borrós§ and Gemma Gabriel†,¶,*

†Instituto de Microelectrónica de Barcelona IMB-CNM (CSIC), Campus Universitat Autònoma de Barcelona, 08193 Cerdanyola del Vallès, Barcelona, Spain

‡PhD in Electrical and Telecommunication Engineering, Universitat Autonoma de Barcelona (UAB)

§ Grup d'Enginyeria de Materials, Institut Químic de Sarrià-Universitat Ramon Llull, vía Augusta 390, 08017 Barcelona, Spain

¶CIBER de Bioingeniería, Biomateriales y Nanomedicina (CIBER-BBN)

AUTHOR INFORMATION

Corresponding Author

*E-mail: gemma.gabriel@imb-cnm.csic.es

Pseudo-reference electrode

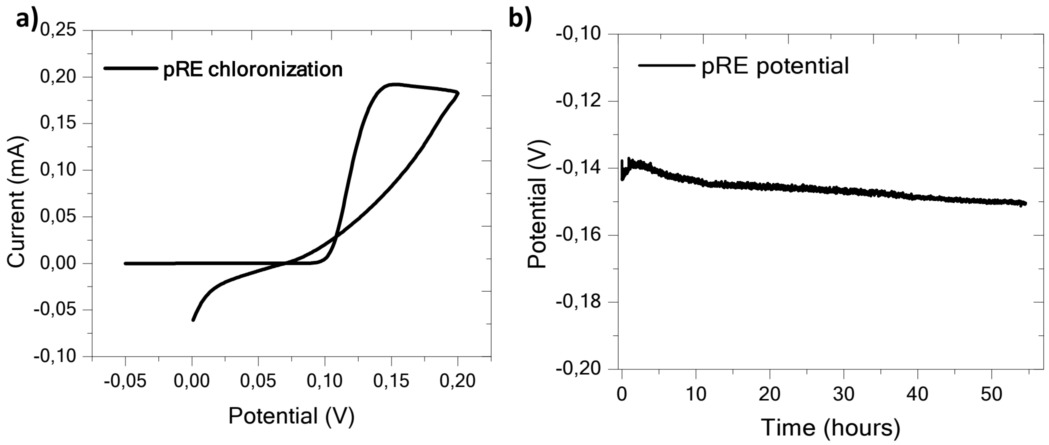

Figure S1. a) CV in HCl 0.1 M at a scan rate of 20 mV/s applied for the chlorinated process of the printed Ag electrode, b) Potential stability in KCl (0.1 M) evaluated against a commercial Ag/AgCl reference electrode (RE) of the developed Ag/AgCl pRE over time.

PANI:PSS and PPy:PSS formulations

Formulations		
PANI:PSS (Mz 70.000) solution diluted 1:1 MQ water	Triton X100 1.5% v/v	
	Triton X100 1.5% v/v	DMSO 5% v/v
	Triton X100 1.5% v/v	Gly 5% v/v
PANI:PSS (Mz 200.000) solution diluted 1:1 MQ water	Triton X100 1.5% v/v	
	Triton X100 1.5% v/v	DMSO 5% v/v
	Triton X100 1.5% v/v	Gly 5% v/v
PPy:PSS (Mz 70.000) solution diluted 1:1 MQ water	Triton X100 1.5% v/v	
	Triton X100 1.5% v/v	DMSO 5% v/v
	Triton X100 1.5% v/v	Gly 5% v/v
PPy:PSS (Mz 200.000) solution diluted 1:1 MQ water	Triton X100 1.5% v/v	
	Triton X100 1.5% v/v	DMSO 5% v/v
	Triton X100 1.5% v/v	Gly 5% v/v

Table S1. Formulations of inkjet printing inks obtained from PANI:PSS / PPy:PSS suspensions

Surface tension of PANI:PSS / PPy:PSS formulations

Formulations			mN/m	Standard deviation
PANI:PSS (Mz 70.000) solution diluted 1:1 MQ water	Triton X100 1.5% v/v		59,7	1,3
	Triton X100 1.5% v/v	DMSO 5% v/v	59,2	1,5
	Triton X100 1.5% v/v	Gly 5% v/v	59,3	1,8
PANI:PSS (Mz 200.000) solution diluted 1:1 MQ water	Triton X100 1.5% v/v		59,7	1,2
	Triton X100 1.5% v/v	DMSO 5% v/v	59,2	1,5
	Triton X100 1.5% v/v	Gly 5% v/v	59,4	2,1
PPy:PSS (Mz 70.000) solution diluted 1:1 MQ water	Triton X100 1.5% v/v		59,7	1,2
	Triton X100 1.5% v/v	DMSO 5% v/v	59,9	2,6
	Triton X100 1.5% v/v	Gly 5% v/v	59,8	1,5
PPy:PSS (Mz 200.000) solution diluted 1:1 MQ water	Triton X100 1.5% v/v		61,4	2,8
	Triton X100 1.5% v/v	DMSO 5% v/v	60,1	1,8
	Triton X100 1.5% v/v	Gly 5% v/v	59,7	1,9
PANI:PSS + PPy:PSS (Mz 70.000)+MQ water (1:1:1)	Triton X100 1.5% v/v	DMSO 5% v/v	60,5	1,6

Table S2. Surface tension of the formulations of inkjet printing inks obtained from PANI:PSS / PPy:PSS suspensions

IR spectra characterization

PANI:PSS and PPy:PSS suspension were characterized using FTIR spectra to validate the obtention of the CPs suspension. For PANI, main PANI bands are present in FTIR (Figure S2 a): 1578 cm^{-1} band correspond to C = C stretching of PANI quinoid groups and 1486 cm^{-1} C = C stretching of benzenoid groups highlighted in the green bar. Also, it is possible to identify the 1294 cm^{-1} band of stretching vibration of secondary

amine C – N band and 830 cm^{-1} band corresponding at C – H out-of -plane deformation vibration of the benzenoid groups[1]. The FTIR also presented the sulfonic acid vibration bands at 1138 and 1110 cm^{-1}, 803 cm^{-1} band corresponding to out-of-plane bending vibration of the C-H band of p-disubstituted benzene ring and 505 cm^{-1} band corresponding to – SO_3H group stretch[1,2]. These bands confirm the presence of a p – substituted benzene group with sulfonic acid in the PANI microparticles.

IR spectre for PPy:PSS 70.000, revealed the principal bands of both compounds as was expected (Figure S2 b). Typical pyrrole ring vibration bands at 1550cm^{-1}, 930cm^{-1} and 770 cm^{-1} along with C – N stretch band (1189 cm^{-1}) and 666 cm^{-1} from primary amine wagging confirms the presence of PPy in the structure[3,4]. The FTIR also presented the sulfonic acid vibration bands at 1033 and 1004 cm^{-1} observed previously in the PANI FTIR (Figure S2 a) confirming the presence of the polyelectrolyte[1,2].

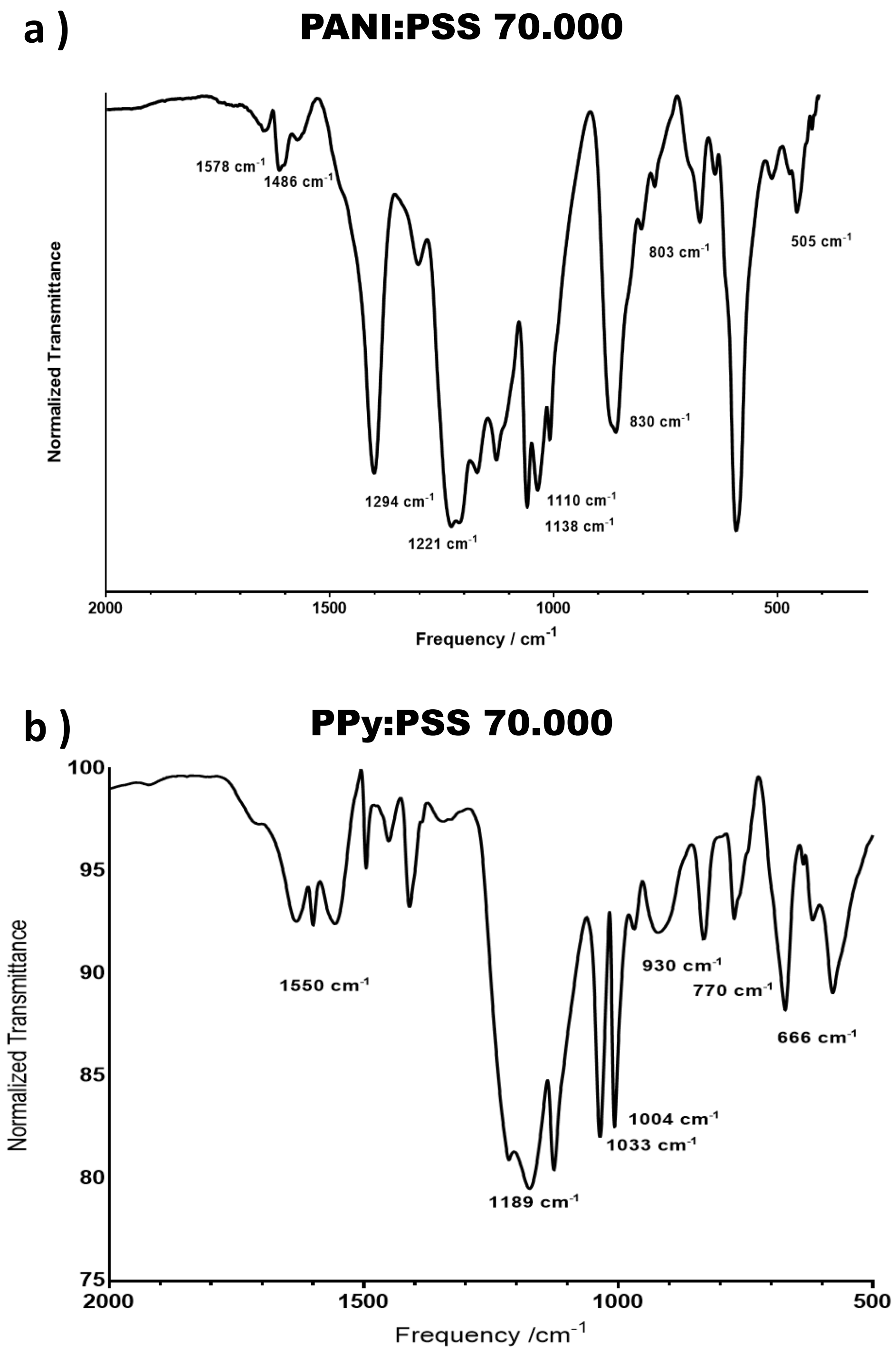

Figure S2. FTIR spectra of PANI:PSS Mw 70.000 (a) and PPy:PSS Mw 70.000 (b).

CP suspensions – pH dependence

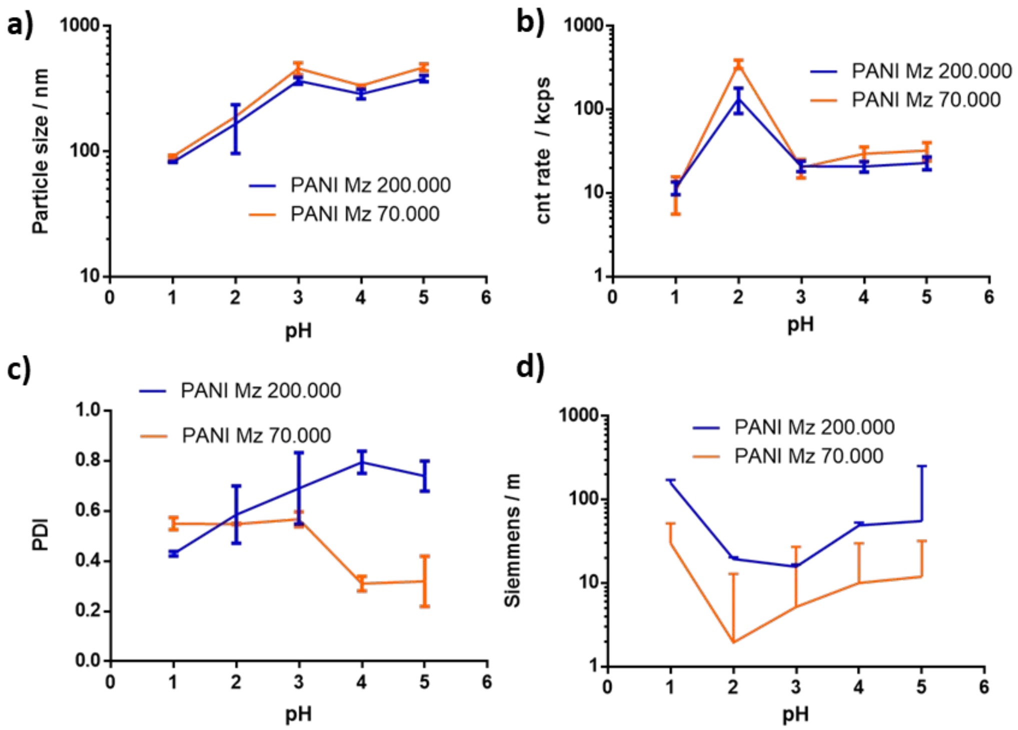

Figure S3. Effect of the pH of the synthesis media on PANI:PSS suspension parameters: Particle size mean (a), count rate of the DLS measurement, indicating number of particles in media (b), Polydispersity of the suspensions (c) and conductivity of the obtained film (d).

DLS characterization

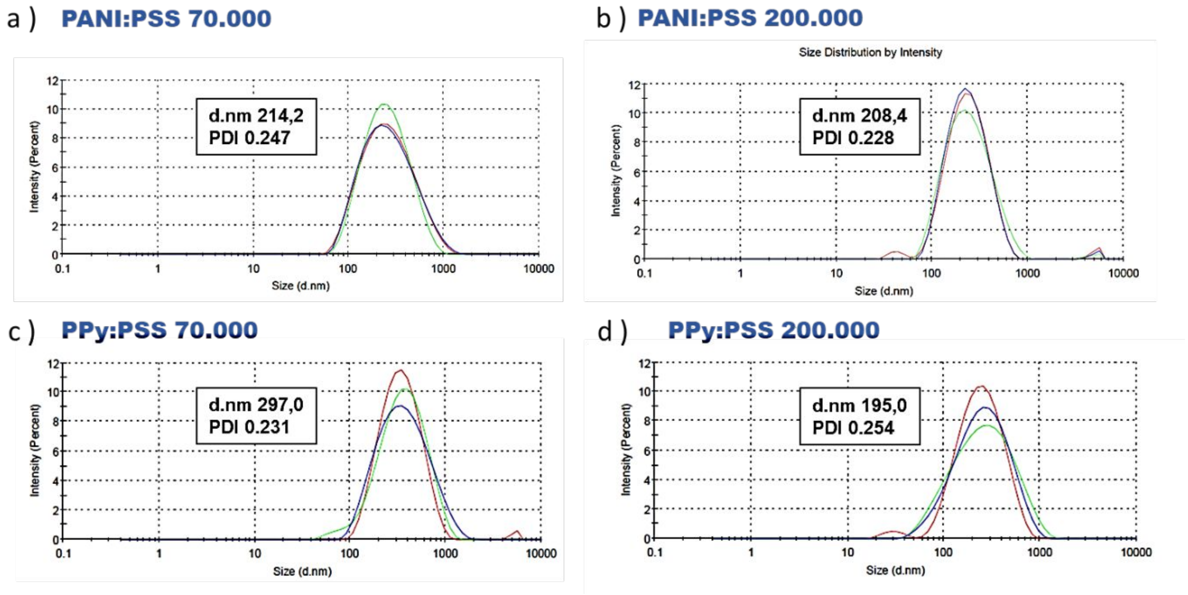

Figure S4. Particle size distribution profile obtained through DLS for CP suspensions after its synthesis: PANI:PSS with PSS molecular weight of Mw 70.000 and 200.000 (a and b) and PPy:PSS with a Mw of 70.000 and 200.000 (c and d).

Zeta potential characterization

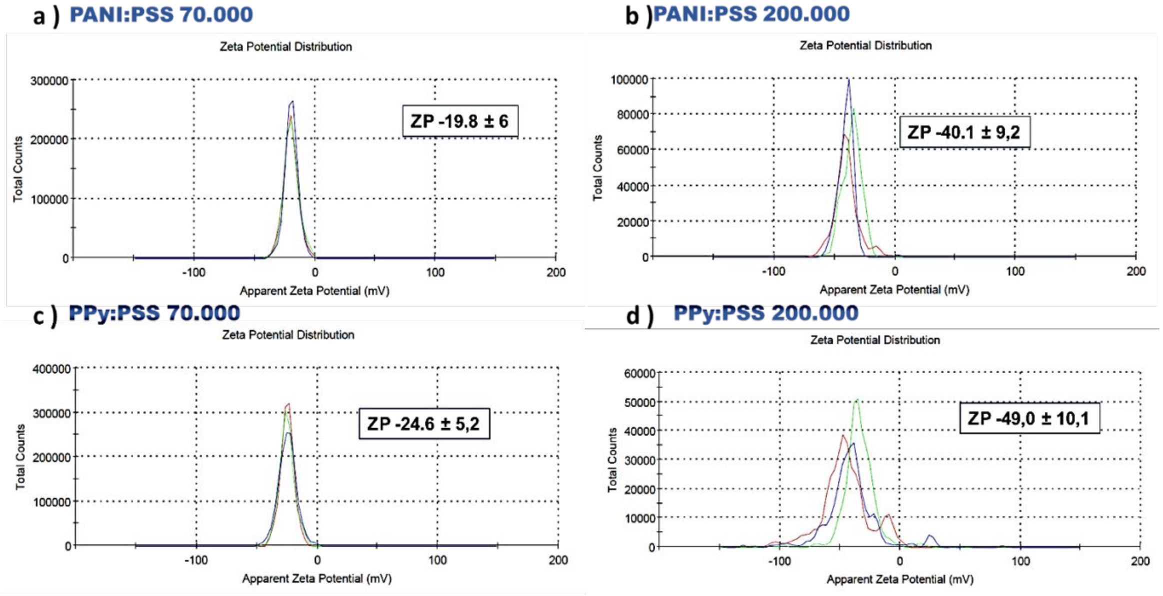

Figure S5. Zeta potential profile obtained through DLS for CP suspensions after its synthesis: PANI:PSS with PSS molecular weight of Mw 70.000 and 200.000 (a and b) and PPy:PSS with a Mw of 70.000 and 200.000 (c and d).

FESEM images

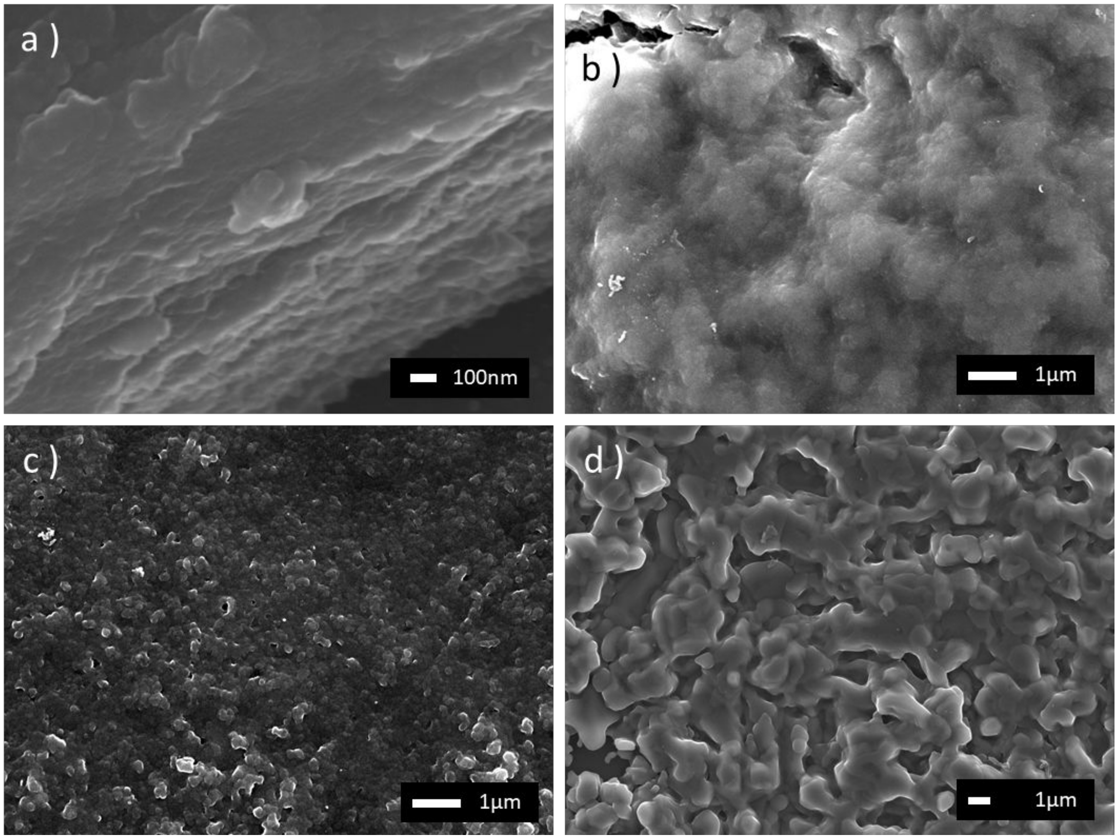

Figure S6. FESEM images of CP suspension films: a) PANI:PSS Mz 70.000, b) PANI:PSS Mz 200.000, c) PPy:PSS Mz 70.000 and d) PPy:PSS Mz 200.000.

Inkjet Printing fabrication

Waveform was develop to fine-tune the drop ejection of the inkjet printheads.

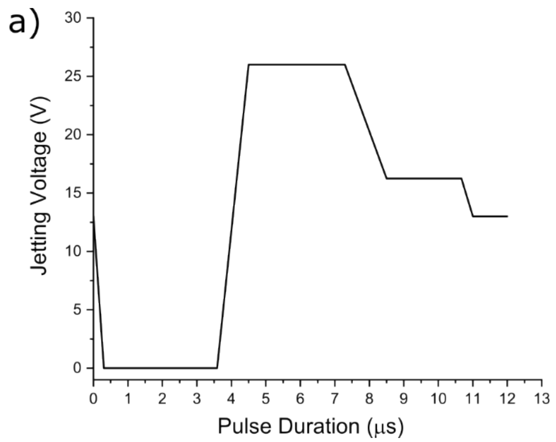

b)

**Print resolution / Drop space**	**2540 dpi / 10 μm**
Maximum jetting frequency	5 KHz
**Maximum Jetting Voltage**	**26 V**
Maximum number of Nozzles	15

Figure S7. Optimized waveform and parameters applied to the piezoelectric transducer of the printhead for the CP inks printing developed to fine-tune the drop ejection of the inkjet printhead.

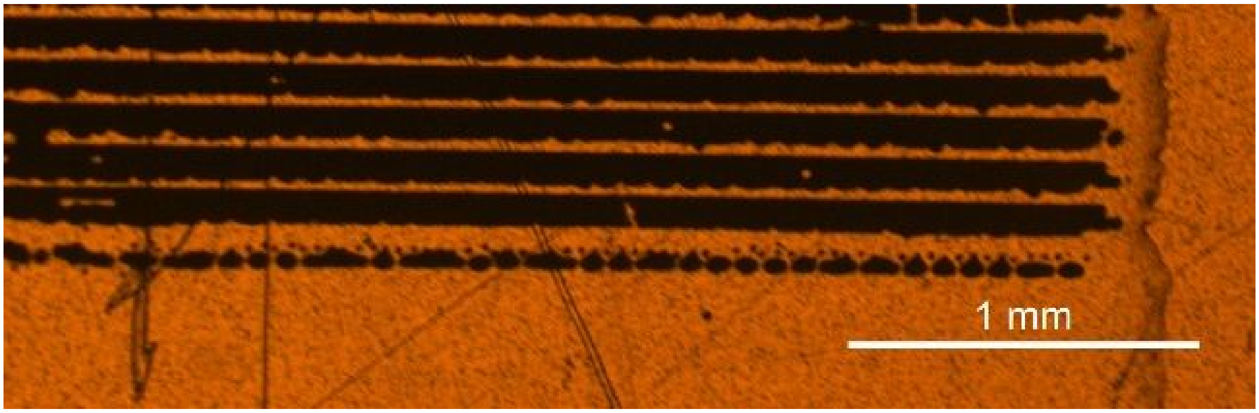

Figure S8. Image of a square printed at DS 10 μm. This DS or higher do not allow the overlap of contiguous printed lines.

Non-adherence of polymeric film

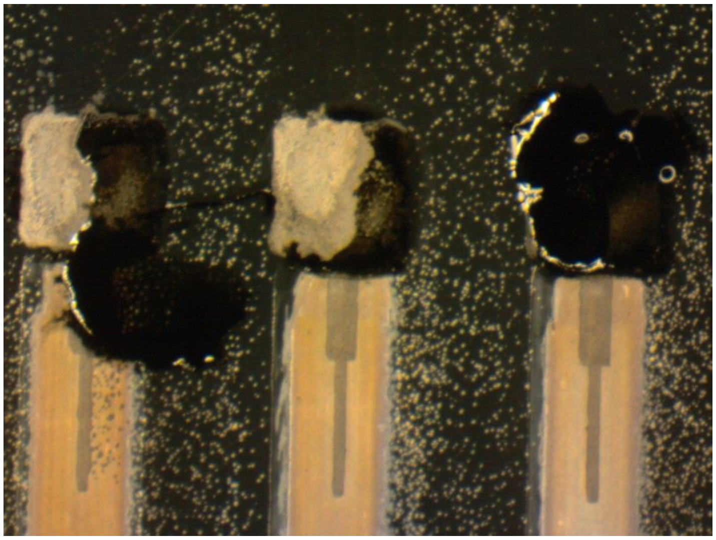

Figure S9. Four layers (4L) of the printed CP produce films that are completely detached when being immersed in an aqueous solution due to an excess of material over the metallic electrode.

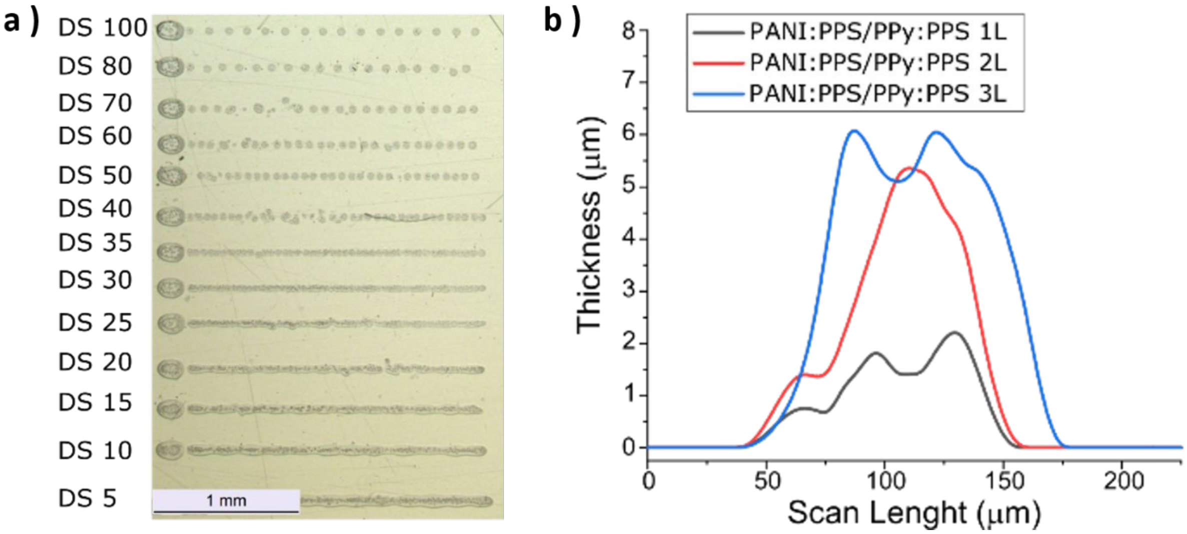

Figure S10. a) Line pattern printed on PEN substrate for PANI:PSS/PPy:PSS specially formulated ink and b) the cross-section profiles for 1L, 2L and 3L.

		Volts		
**pH**	**Electrode 1**	**Electrode 2**	**Electrode 3**	**CV (%)**
**10**	-0,11235	-0,11098	-0,11123	0,65642434
**9**	-0,03994	-0,03894	-0,03888	1,52130896
**8**	0,07205	0,07296	0,07307	0,77200719
**7**	0,15627	0,15694	0,15701	0,26191204
**6**	0,23104	0,23081	0,23082	0,05691322
**5**	0,29630	0,29583	0,29582	0,09317697
**4**	0,37870	0,37715	0,37714	0,23830115
**3**	0,45789	0,45899	0,45800	0,13207177

Table S3. Variation coefficient of calibration in figure 6 with the PANI:PSS/PPy:PSS ink.

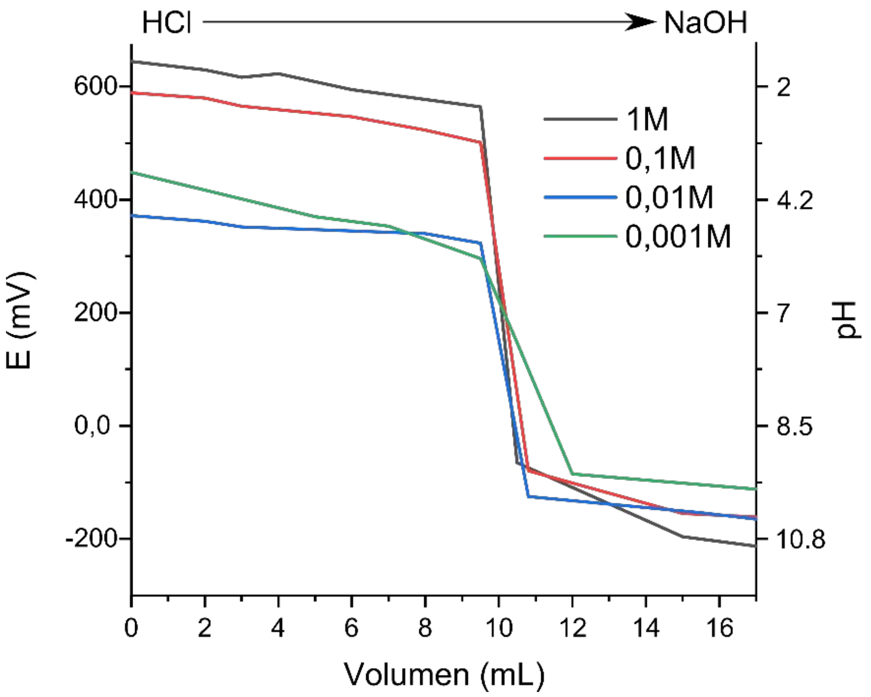

Figure S11. Titration of strong acid with strong base: Potentiometric pH titration curves for HCl and NaOH at the indicated molarity

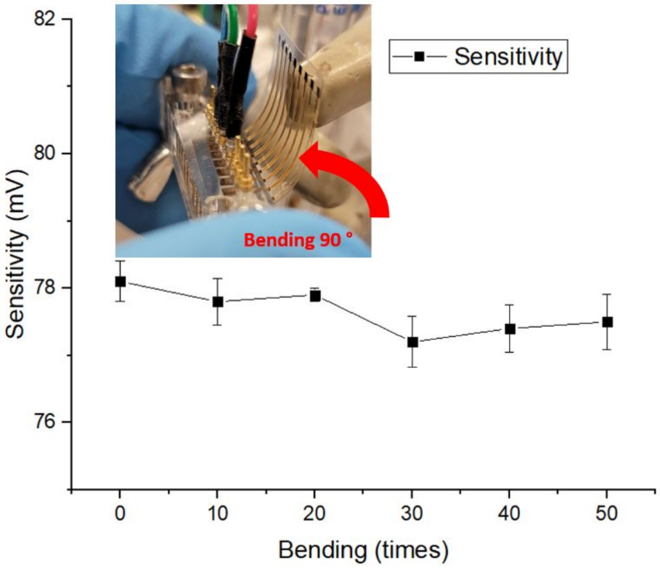

Figure S12. Evolution of the pH sensor sensitivity after a simple bending test where the electrode platform is cyclically bended 90 °.

[1] A. Manzoli, F. M. Shimizu, L. A. Mercante, E. C. Paris, O. N. O. Jr, S. Correa, L. H. C. Mattoso, *Phys. Chem. Chem. Phys.* **2014**, *16*, 24275.

[2] W. Hu, S. Chen, Z. Yang, L. Liu, H. Wang, *J. Phys. Chem. B* **2011**, 8453.

[3] J. Y. Lee, J.-W. Lee, C. E. Schmidt, *J. R. Soc. Interface* **2009**, *6*, 801.

[4] T. Dai, X. Yang, Y. Lu, *Mater. Lett.* **2007**, *61*, 3142.
